# Metformin reverses 5-FU resistance induced by radiotherapy through mediating folate metabolism in colorectal cancer

**DOI:** 10.1186/s10020-025-01206-5

**Published:** 2025-05-21

**Authors:** Shuxuan Wang, Yanyan Lin, Qianqian Zhao, Huanliang Chen, Shisuo Du, Zhaochong Zeng

**Affiliations:** 1 Department of Radiation Oncology, Zhongshan Hospital, Fudan University, Shanghai, 200032 China; 2 Cancer Center, Zhongshan Hospital, Fudan University, Shanghai, 200032 China

**Keywords:** Irradiation, 5-FU, Histocompatibility minor 13, Metformin, Folate metabolism

## Abstract

**Purpose:**

Radiation therapy has revolutionized the treatment of primary or liver metastases in colorectal cancer (CRC). In colorectal cancer, conventional fractionation (1.8 ~ 2.0 Gy daily) is typically used for treatment. Nevertheless, there is a paucity of research investigating the potential implications of radiation therapy-induced alterations in the expression levels of regulatory genes on resistance to chemotherapy agents. Herein, we explored the mechanism by which conventional fractionation drives 5-fluorouracil (5-FU) resistance and metformin (Met) rescued 5-FU resistance in CRC.

**Methods and materials:**

RNA sequencing, differential genes expression analysis was performed to identify the 5-FU resistance genes after irradiation (according to the convention of cell irradiation, 2 Gy × 8 scheme was selected). Drug sensitivity assay, immunofluorescence staining, folate analogs concentration measurement was used to explore the biological function of histocompatibility minor 13 (HM13) and γ-Glutamyl Hydrolase (GGH). Combined chemosensitivity test and xenograft mouse model has been used to gain insights into the underlying clinical value of the combination of 5-FU and Met.

**Results:**

The conventional fractionation scheme (2 Gy × 8) induced resistance to 5-FU in the CRC cell line HCT-15, accompanied by an elevated RNA expression level of peptidase HM13. Mechanistically, the increased expression of HM13 caused an abnormal shearing of the N-terminal signal peptide of γ-Glutamyl Hydrolase (GGH), which resulted in decreased intracellular content of 5, 10-methylenetetrahydrofolate (5,10-CH_2_-THF).

**Conclusion:**

We revealed a new mechanism of 5-FU resistance induced by irradiated with 2 Gy × 8 through the HM13-GGH-5,10-CH_2_-THF axis. The synergistic effect of Met and 5-FU can rescue 5-FU resistance after conventional fractionated irradiation. In summary, this work will help to reveal the mechanisms of IR-induced 5-FU resistance, which is important for finding new therapeutic targets and improving the efficacy of chemotherapy regimens after radiotherapy.

**Supplementary Information:**

The online version contains supplementary material available at 10.1186/s10020-025-01206-5.

## Introduction

Colorectal cancer (CRC) is a common cancer of the digestive tract that affects older people (Dekker et al. 2019). The prognosis of CRC has currently been improved following the combination treatment of surgery and neoadjuvant chemotherapy or radiation treatments (Dekker et al. 2019). However, CRC remains the second greatest cause of cancer-related death globally (Dekker et al. 2019). Locally advanced CRC is treated with a mix of chemotherapy, surgery, and radiotherapy. Radiotherapy primarily exerts its lethal effects by activating the immune system, altering the tumor microenvironment, modifying cellular metabolic cycles, and impacting DNA damage repair (DDR) mechanisms (McLaughlin et al. 2020; Du et al. 2021). Research indicates that there are significant differences in gene expression levels after radiotherapy (Tsai et al. 2007; Haviland et al. 2013).

The current standard therapy is based on fluorouracil as monotherapy or combination therapy. Clinically, this class of drugs includes 5-FU, Capecitabine, Tegorio, etc. 5-FU, as a classic drug in the chemotherapy regimen for CRC, has well-documented efficacy in continuous low-dose infusion therapy (Dekker et al. 2019). However, the current study revealed that adjuvant chemotherapy with 5-FU + leucovorin (LV) after radiotherapy does not enhance the prognosis of locally advanced CRC (Bosset et al. 2014; Sainato et al. 2014). Conversely, recent research has shown that 5-FU sensitizes cells to radiation by regulating multiple biological processes which is in sharp contrast to the poor efficacy of 5-FU after radiotherapy (Tsai et al. 2007; Tang et al. 2017). However, the reason for the lack of improvement in prognosis when using 5-FU after radiotherapy remains unknown.

5-FU is an uracil analog, it metabolizes within the cell to 5-fluoro-2’-deoxyuridylate monophosphate (FdUMP) and forms a ternary complex with 5,10-CH_2_-THF and thymidylate synthase (TS), which blocks 2′-deoxyuridine-5′-monophosphate (dUMP) from accessing the nucleotide binding site of TS, resulting in an imbalance in the deoxynucleotide triphosphate (dNTP) pool (Longley et al. 2003). The depletion of deoxythymidine monophosphate (dTMP) promotes the consumption of deoxythymidine triphosphate (dTTP), and disrupts the balance of other dNTPs. This imbalance in the deoxyuridine triphosphate (dNTP) pool impairs DNA synthesis and DDR. The increased deoxyuridine triphosphate (dUTP) and 5-fluoro-2’-deoxyuridine triphosphate (5-FdUTP) promote uracil misincorporation into DNA (Longley et al. 2003).

Since the application of 5-FU to cancer treatment, numerous studies studies have focused on the cause of 5-FU resistance, with any alteration in the constituents of the ternary complex potentially leading to the development of drug resistance (Marsh et al. 2001). Emerging evidence has increasingly elucidated the critical interplay between metabolic reprogramming, DNA damage repair, and chemoresistance mechanisms, DNA damage repair, and drug resistance (Acharya et al. 2024). Therefore, we hypothesized that radiotherapy could modulate cellular metabolic pathways and thereby affect chemosensitivity to 5-FU. Depending on how the radiation dose is divided, radiotherapy fractionation schemes are divided into Hypofractionated radiotherapy (HFRT) and Conventional fractionation radiotherapy (CFRT); CFRT (1.8 ~ 2.0 Gy per day) is based on the radiobiological characteristics of tumor cells and takes advantage of the repair capacity of normal tissues to reduce toxicity during a long treatment process while ensuring continuous killing of tumor cells (Ahmad et al. 2012). In the treatment of CRC, conventional fractionation scheme is more widely used. Therefore, we investigated the therapeutic effect of 5-FU after the conventional fractionation schemes and the underlying mechanisms of 5-FU resistance.

Metformin is the first-line medication to treat type 2 diabetes mellitus (T2DM) (Jara and López-Muñoz 2015). Furthermore, increasing evidence points towards other diseases that might also have an important role, including cancer, age-related diseases and inflammatory diseases (Jara and López-Muñoz 2015). Metformin has been proven in studies to be effective as an adjuvant treatment for CRC (Coyle et al. 2016). And metformin has been referred to as an “antimetabolite” by certain researchers, implying that it can influence one-carbon metabolism. However, the particular effects of metformin on folate metabolites are unclear.

In our study, using RNA sequencing analysis of HCT-15 cells under different treatment conditions, we screened HM13 that was most upregulated after radiotherapy. Based on the biological characteristics of HM13 as a signal peptide peptidase and Effect of radiotherapy on folate circulation, we identified the only GGH protein carrying an N-terminal signal peptide from the 5-FU metabolic pathway and key enzymes of the folate cycle. And the conventional fractionation scheme (2 Gy × 8) contributed to abnormal expression of HM13, resulting in premature cleavage of the signal peptide of GGH, which causes GGH to remain in the cytoplasm, resulting in reduced levels of the active 5,10-CH_2_-THF. Consequently, the ternary complex composed of 5,10-CH_2_-THF and TS decreases, which eventually leads to 5-FU drug resistance in CRC patients. Based on the fact that metformin can mediate folate circulation, we chose metformin to further explore, and found that the “anti-metabolic effect” of metformin could enhance the cytotoxic effect of 5-FU after radiotherapy, and rescue the 5-FU resistance caused by radiotherapy.

In summary, we discussed potential reasons for the 5-FU resistance following radiotherapy. and revealed that the HM13-GGH-5,10-CH_2_-THF axis can influence the cytotoxicity of 5-FU. We also proposed a protocol for combination therapy of Met with 5-FU to overcome 5-fu resistance. Our findings additionally advance our understanding of the mechanism of chemotherapy resistance after radiotherapy, but also provide an important theoretical foundation for optimizing clinical radiotherapy regimens and selecting adjuvant chemotherapy after radiotherapy.

## Materials and methods

### Cell culture

Human CRC cell lines HCT-15 (RRID: CVCL_0292) and HT-29 (RRID: CVCL_0320), and human embryonic kidney cell line 293T (HEK 293T (RRID: CVCL_0063)), were purchased from the Institute of Biochemistry and Cell Biology, Chinese Academy of Sciences (Shanghai, China). HCT-15 cells were cultured in RPMI 1640 medium (Gibco, USA) supplemented with 10% fetal bovine serum (FBS) (Sigma, Germany), 100 U/ml penicillin-streptomycin (NCM Biotech, China). HT-29 cells were cultured in McCoy’s 5 A medium (Gibco, USA) with 10% FBS (Sigma, Germany), 100 U/ml penicillin-streptomycin (NCM Biotech, China). The 293T cells were cultured in DMEM medium supplemented with 10% FBS (Sigma, Germany), 100 U/ml penicillin-streptomycin (NCM Biotech, China). All cells were incubated at 37 °C in a humidified atmosphere containing 5% CO_2_. Mycoplasma contamination was regularly tested in all cell cultures. All cell lines were authenticated using short tandem repeat (STR) profiling within the last 3 years. All experiments were performed with mycoplasma-free cells.

Construction of the HCT-15/5-FU and HCT-15/L-OHP Cell Line. The human CRC 5-FU-resistant cell line HCT-15/5-FU was established using a continuous stepwise drug concentration increment method with 5-FU. This cell line stably grows in RPMI 1640 medium containing 20 µM 5-FU, exhibiting RI (resistance index) of 10.4. The human CRC L-OHP-resistant cell line HCT-15/L-OHP was established using a continuous stepwise drug concentration increment method with L-OHP. This cell line stably grows in RPMI 1640 medium containing 20 µM 5-FU, exhibiting RI (resistance index) of 12.5.

### mRNA sequencing experimental method

Detailed mRNA sequencing experimental method is available in the Supplemental Materials.

The sequencing coverage and quality statistics for each sample are summarized in Supplementary Table S1.

### Construction of overexpression and knockdown cell lines

The designed of sgRNA sequences was based on the protocol from Zhang’s laboratory, with the following target sequences for CRISPR-Cas9 single-guide RNAs (sgRNAs):

sgHM13: 5’- GATGTCAAAGCGCAGCAGCA-3’.

sgGGH-1: 5’- TGCGTCCTATGTAAAGTACT-3’.

sgGGH-2: 5’- TGACTGCCAATTTCCATAAG-3’.

We used CRISPR-Cas9 system to knockout HM13 and GGH genes. The LentiCRISPR v2 plasmid was used to construct vectors along with packaging plasmids, and 293T cells were infected. HCT-15 and HT-29 cells were infected, and 48 h post-infection, cells were selected using 1 µg/ml puromycin.

The coding sequences of HM13, GGH, and the GGH mutant lacking the N-terminal signal peptide were cloned into the pGV348-HA vector. Following lentiviral packaging, the constructs were used to infect HCT-15 and HT-29 cells. Infected cells were selected with 1 µg/ml puromycin 48 h post-infection.

### RNA extraction and reverse transcription quantitative PCR analysis

Total RNA was extracted by MolPure^®^ Cell RNA Kit (Cat. 19231, Yeasen, China) and reverse transcribed (RT) to cDNA using Hifair^®^ AdvanceFast One-step RT-gDNA Digestion SuperMix for qPCR (Cat. 11151, Yeasen, China). Quantitative PCR (qPCR) reactions were carried out with qPCR SYBR Green Master Mix (No Rox) (Cat. 11201, Yeasen, China). Relative mRNA expression levels were normalized to ACTB.

Primers used included:

HM13,

F-CTGGTACCTGCTGAGGAAGC

R-GGTGCCAAATACCCAGAAGA

GGH,

F_−ΔN_-TCCTTTTCCCTGGAGGAAGT

F-TCTGGAACATTCTGCTGTGC

R-GTGCTGGGCCTGCTACTCT

ACTB,

F-CATCCGCAAAGACCTGTACG

R-CCTGCTTGCTGATCCACATC

### Western blot analysis

Primary antibodies used for western blot were: HM13 (ab190253, Abcam, UK), GGH (13264-1-AP, Proteintech, USA), GAPDH (60004-1-Ig, Proteintech, USA), ACTIN (66009-1-Ig, Proteintech, USA), LAMP-2 (SC-18822, Santa Cruz, USA), Tubulin (10068-1-AP, Proteintech, USA). Secondary antibodies used for western blot were: HRP-conjugated Goat anti-Rabbit/Mouse IgG (A0208/A0216, Beyotime, China). Enhanced chemiluminescence (ECL) reagent (WBKLS0500, Millipore, USA). The band quantification was measured by ImageJ software (NIH).

### Cell viability assay

5,000 cells were seeded in each well of 96-well plates and treated for 72 h with gradient concentration of 5-FU, and cell viability was measured by the CellTiter-Glo Luminescent Kit (Cat. G7573, Promega, USA). The half-maximal inhibitory concentration (IC_50_) was then calculated by GraphPad Prism 8.0.2. Each experiment was performed in triplicate, and results were expressed as mean ± SD.

To determine the combined drug treatment effects and calculate synergy efficiency, cells were treated with 5-FU at concentrations of 0, 1, 3, 10, 30, 100, 300 and 1000 µM, with or without Met at concentrations of 0, 0.5, 1, 2, 5, 10 and 20 mM. The HSA mode synergy score was calculated using the SynergyFinder web application to evaluate the synergistic effects.

### Immunofluorescence staining

HCT-15 cells subjected to irradiation treatment were cultured on coverslips, then fixed with ice-cold methanol for 5 min, and permeabilized with 0.1% Triton X-100. The cells were blocked with 1% bovine serum albumin in PBS for 1 h, followed by antibody staining (anti-GGH antibody, 1:200; anti-LAMP-2 antibody, 1:200). Alexa Fluor 488-conjugated goat anti-mouse IgG and Alexa Fluor 647-conjugated goat anti-mouse IgG were used as secondary antibodies. After washing with PBS, the cells were mounted with DAPI-containing ProLong Gold antifade reagent. Analysis was performed using an SP5 confocal microscope.

### Cell irradiation experiment

Cells in the logarithmic growth phase were irradiated using the ONCOR™ linear accelerator (Siemens, Munich, Germany) with 2 Gy × 8 of X-ray irradiation.

### ELISA detection

The Methionine, SAH, SAM and Homocysteine content were measured using the Methionine ELISA kit (QEK13852, Biogradetech, USA), SAH ELISA kit (QEK04333, Biogradetech, USA), SAM ELISA kit (QEK01036, Biogradetech, USA) and Homocysteine ELISA kit (CSB-E13814h, CUSABIO, China), following the manufacturer’s instructions.

### Signal peptidase site prediction

Key gene clusters in the 5-FU metabolic and folate metabolism pathway were selected, and predictions of signal peptidase sites were analyzed using the SignalP 6.0 web application: all protein sequences were imported into SignalP 6.0 (fasta format) and analyzed under the following conditions: Organism: Eukarya; Output format: Long output; Model mode: Fast. Analysis of signal peptide positions (CS positions) based on the output prediction results (Teufel et al. 2022).

### Intracellular folate and folate analogs concentration measurement

Cells were collected and resuspended in 500 µL of 95% methanol/extraction buffer (75 mM KH_2_PO_4_, 0.4 M ascorbic acid, 0.8% 2-mercaptoethanol, pH 6.0). After vortexing for 1 min, the samples were ground at 65 Hz for 300 s and sonicated at 4 °C for 30 min, then allowed to stand for 1 h. The supernatant was dried by a rotary evaporator after centrifugation at 12,000 × rpm for 15 min at 4. The residue was resuspended in 100 µL of 95% methanol/extraction buffer. And the supernatant was measured by liquid chromatography on Waters Acquity UPLC and mass spectrometry by AB SCIEX 5500 Qtrap-MS. Folate, 5,10-CH_2_-THF and 5-methyl-tetrahydrofolate (5-CH_3_-THF) concentrations were calculated using MultiQuant software and standard curves.

The concentration of folic acid analogue polyglutamated metabolite was determined using peak area analysis, following the protocol by Lee C. Garratt (Garratt et al. 2005).

### Xenograft mouse model

6-8-week-old male nude mice were used and housed in a pathogen-free environment. HCT-15 cells (5 × 10^6^) were subcutaneously implanted into the mice’s right flank., mice were irradiated at 2 Gy × 8 when tumors reached a volume of 100 mm (Du et al. 2021).which were then randomly separated into four groups (DMSO, Met, 5-FU, Met + 5-FU). 5-FU (20 mg/kg), Met (100 mg/kg), or both drugs combined were intraperitoneally injected into mice every other day after irradiation, the tumor volumes were monitored with calipers every two day. And the tumor volumes were calculated by the formula: 1/2 length × width^2^. Tumors were harvested for western blot analysis on day 30 post-irradiation (the tumor volume < 1000 mm (Du et al. 2021).

### Statistical analysis

The statistical analysis was conducted by GraphPad Prism 8.0 software. Data were shown as the mean ± SD, and three independent experiments were performed. In the xenograft mouse model, each experimental group comprised 6 mice, to mitigate potential outliers, the maximum and minimum tumor volume values within each group were excluded prior to statistical analysis. A two-tailed Student’s t-test was used to compare group means. Survival curve analysis was analyzed using two-way ANOVA, *P < 0.05, **P < 0.01, ***P < 0.001, ****P < 0.0001.

## Results

### IR significantly up-regulated the mRNA expression of HM13 in CRC

**Fig. 1:**
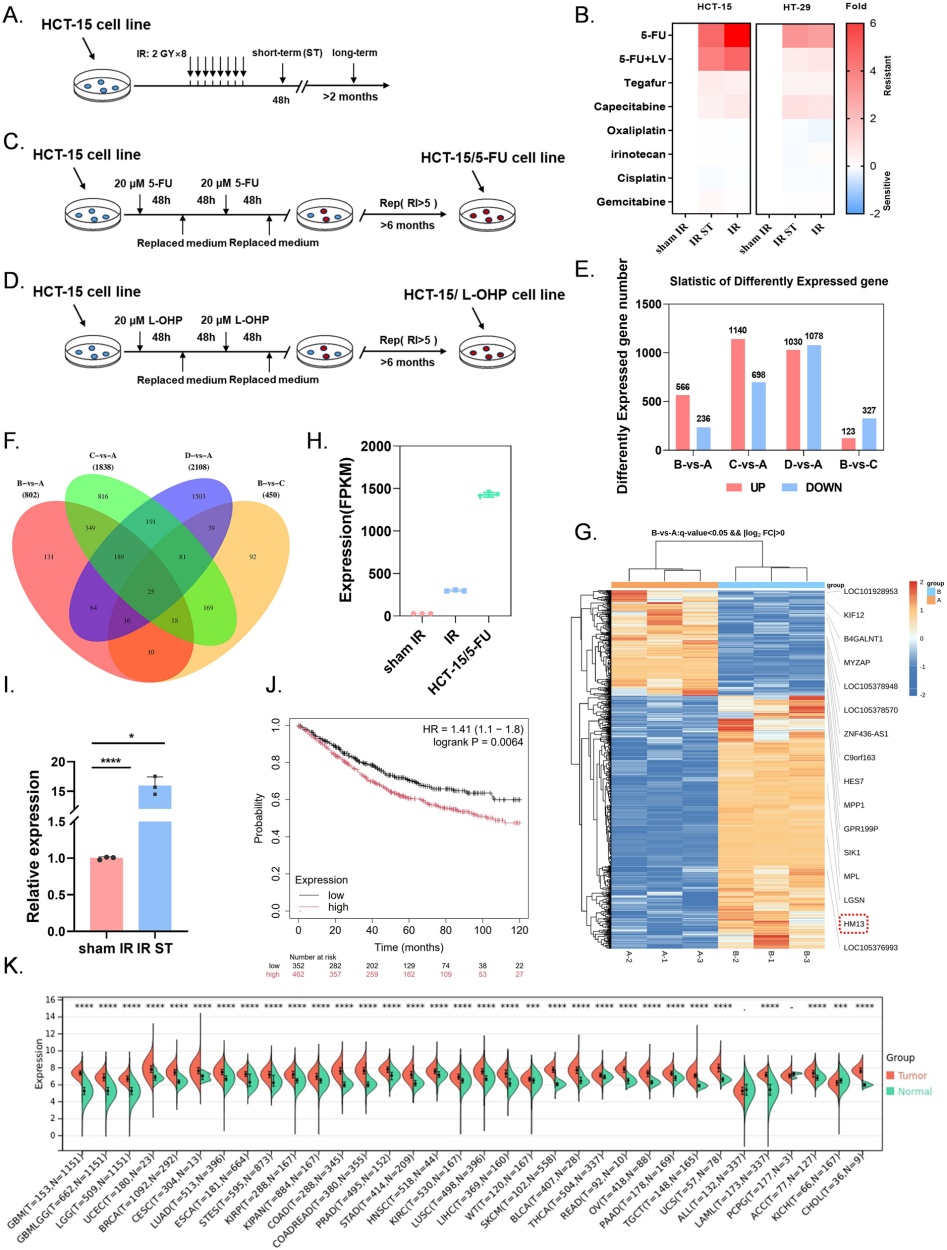
IR significantly up-regulated the mRNA expression of HM13 in CRC (**A**) Scheme for the treatment of the HCT-15 cell line. HCT-15 cells were subjected to irradiation with 2 Gy × 8. Cells were collected for analysis at two time points: after short-term culture (48 h post-irradiation, short-term: 2 Gy × 8 ST) and after long-term culture (2 months post-irradiation, long-term: 2 Gy × 8). (**B**) The heatmap based on the IC_50_ of response to 5-FU, 5-FU + LV, Tegafur, Capecitabine, Oxaliplatin, Irinotecan, Cisplatin, and Gemcitabine in various HCT-15 (Left) or HT-29 (Right) cells. (**C-D**) Scheme of 5-FU-resistant (**C**) or L-OHP-resistant (**D**) cell line construction. HCT-15 cells were cultured in medium containing 20 µM 5-FU (**C**) or 20 µM L-OHP (**D**). The medium was replaced with 5-FU-free medium or L-OHP-free medium every 48 h for buffering. This process was repeated continuously for 6 months, and RI (5-FU-resistant) = 10.4. RI (L-OHP-resistant) = 12.5. (**E**) Statistic of DEGs using RNA-seq data from four experimental groups, **A**: sham IR group (0 Gy); **B**: IR group (2 Gy × 8); **C**: L-OHP-resistant group; **D**: 5-FU resistance group. And q-value < 0.05, |log^2^FC| > 1.0. (**F**). Venn of shared and specific DEGs between different groups. (**G**). The heatmap based on the RNA-SEQ results of 1B. (**H**). The mRNA expression level of HM13 in sham IR, IR and HCT-15/5-FU groups. (**I**). The mRNA expression level of HM13 in sham IR and 48 h post-IR. (**J**). Survival analysis using Kaplan-Meier Plotter web software. Kaplan-Meier Plot analysis demonstrating the prognostic association of HM13 expression with OS in patients with CRC. The log rank test was performed to compare high and low HM13 expression groups. Univariate Cox regression was used to calculate the hazard ratio (HR) and its related 95% confidence interval. (**K**). Differential expression of HM13 across pan-cancer analyzed using SangerBox. *P < 0.05, ****P < 0.0001

We constructed a CRC cell model of conventional fractionated sheme of radiotherapy (2 Gy × 8 regimens) (Fig. 1A). Drug resistance testing with commonly used chemotherapeutic drugs conducted after irradiation revealed that the half-maximal inhibitory concentration (IC_50_) value of 5-FU and other similar metabolic drugs increased significantly at 48 h post-irradiation (short-term, ST) and after long-term culture (over 2 months), the inhibition rate of other commonly used chemotherapy drugs did not change significantly (Fig. 1A-B, Fig S1A-C). And 5-FU-resistant CRC cell lines may develop cross-resistance to oxaliplatin (L-OHP‌‌), we constructed L-OHP‌‌ resistant cell lines (HCT-15/L-OHP‌‌) and 5-FU resistant cell lines (HCT-15/5-FU) of CRC, and performed RNA-seq to identify the resistance genes responsible for 5-FU resistance only affected by radiotherapy (Fig. 1C-D, Fig S1A-C).

To explore the potential contribution of irradiation in 5-FU resistance, we initially examined the differential gene expression by using RNA sequencing (RNA-seq) analysis in CRC cell lines irradiated (2 Gy × 8) and on 5-FU or L-OHP resistance cell lines: sham IR control (Group A), IR (Group B), L-OHP-resistant cell (Group C), and 5-FU-resistant cells (Group D).

We identified 566 genes (increased) and 236 genes (decreased) after IR (Fig. 1E). The Venn diagram was used to show the common differentially expressed genes (DEGs) between B and A, B and C, D and A. 41 (16 + 25) genes were found. (Fig. 1F). To focus on specific effects of 5-FU resistance (L-OHP cross resistance was excluded) caused by IR, we excluded 25 DEGs for A vs. D, resulting in 16 unique genes that were annotated by heat maps. (Fig. 1F-G). The mRNA level of HM13 increased the most in IR group compared with the sham IR group (H). HM13 is an important signal peptide peptidase that affects the maturation and activity of many proteins with N-terminal signal peptides, and its critical involvement in metabolic cycles, we selected HM13 for further investigation (Mentrup et al. 2020). In addition, the mRNA expression level of HM13 was significantly up-regulated in IR ST group, IR group and the 5-FU-resistant group, which is a potential 5-FU resistance gene (Fig. 1H-I).

Considering the up-regulated expression of HM13 in CRC, we further investigated its relationship with the prognosis of CRC using the Kaplan-Meier plotter (Gyorffy 2024). The HM13 high expression group had shorter overall survival (OS) than the low expression group (hazard ratio = 1.41, *P* = 0.0064) (Fig. 1J). We discovered that HM13 expression was remarkably upregulated in most cancer types by TCGA database and GTEx database analyzing with the SangerBox 3.0 web software (Fig. 1K) (Shen et al. 2022).

### The increase of HM13 expression level elicits the 5-FU-resistance after IR

**Fig. 2:**
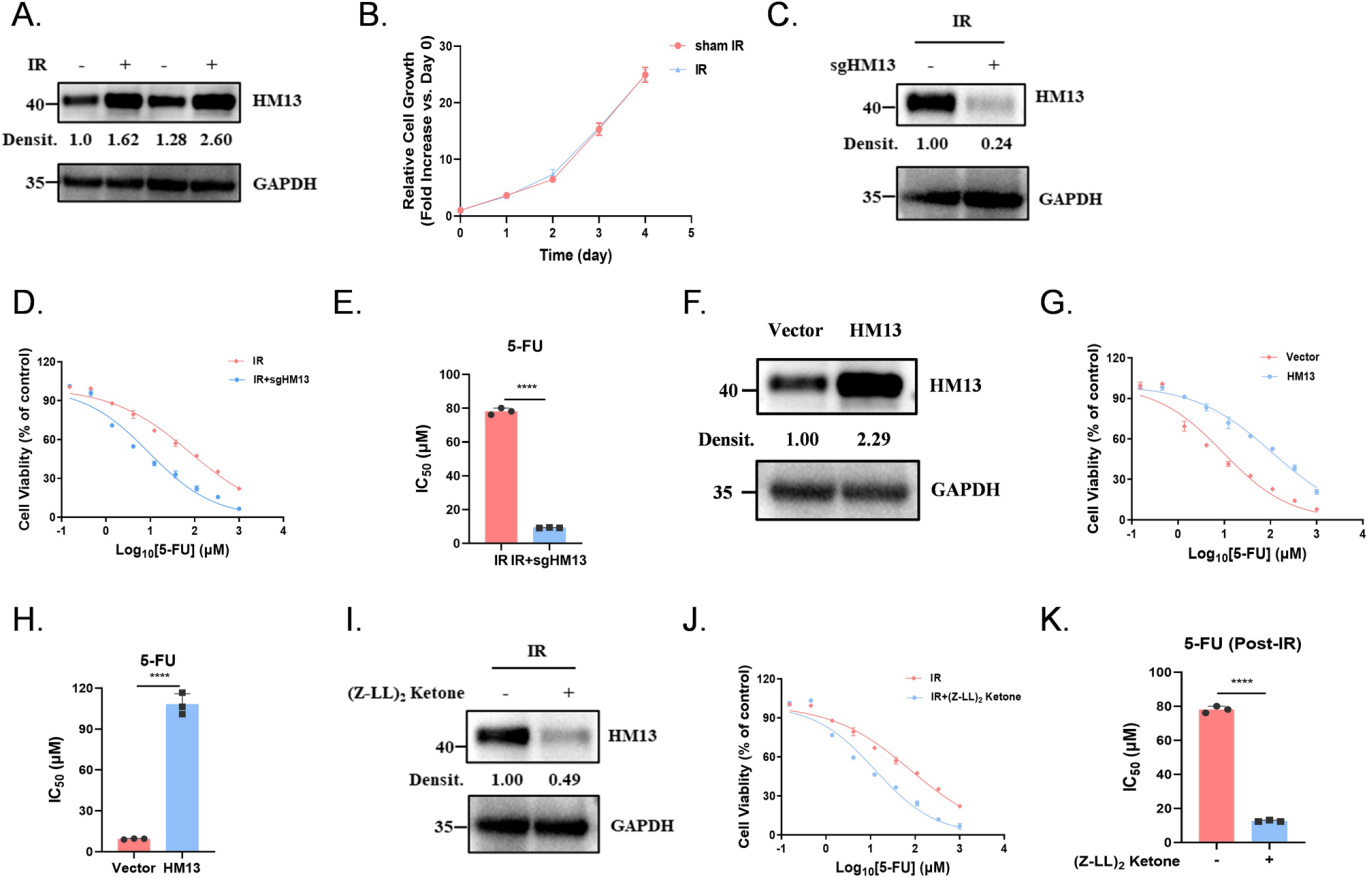
The increase of HM13 expression level elicits the 5-FU-resistance after IR. (**A**) Endogenous HM13 protein expression in IR group and sham IR group. (**B**) Growth curves of IR groups and sham IR groups. The means ± SD are used to display the data. (**C**) Endogenous HM13 protein expression in HCT-15 cells. (**D-E**) Cell viability with 5-FU exposure in mock and sgHM13 HCT-15 cells by IR, the dose–response curve: **D**; the bar graph of IC_50_ values: **E**. (**F**) Overexpression of HM13 in HCT-15 cells. (**G-H**) Cell viability with 5-FU exposure in parental and exogenous overexpression of HM13 HCT-5 cells, the dose–response curve: **G**; the bar graph of IC_50_ values: **H**. (**I**) Endogenous HM13 protein expression in irradiated HCT-15 cells (2 Gy × 8) exposure to the inhibitor of HM13, (Z-LL)_2_ Ketone. (**J-K**) Cell viability with 5-FU exposure in HM13-inhibited HCT-15 cells, the dose–response curve: **J**; the bar graph of IC_50_ values: **K**. All figures were representatives of three independent experiments. Error bars represent the SD. ****P < 0.0001, two-tailed Student’s t-test

Our result showed that when exposed to IR, both short-term and long-term HM13 protein expression levels increased (Fig. 2A). However, the irradiation only affected the cell proliferation level in the short term. (Fig. 2B, S2A).

Additionally, HM13 knockout partially rescued the resistance to 5-FU induced by IR (Fig. 2C-E, S2B-C). And overexpression of HM13 in sham IR also resulted in the development of 5-FU resistance (Fig. 2F-H, S2D-E). Thus, we propose that increased HM13 protein expression is a critical factor in the development of 5-FU resistance.

To further elucidate the impact of HM13 protein on 5-FU resistance, we used the HM13 inhibitor (Z-LL)_2_ Ketone. Similar to the results in Fig. 2F, the addition of (Z-LL)_2_ Ketone to inhibit HM13 protein function after IR partially rescued the cells from 5-FU resistance (Fig. 2I-K, S2F-G). Overall, the results show that that HM13 knockout does not sensitize cells to 5-FU, only shows the resistance caused by increased expression.

**HM13 cleaves the signal peptide**,** resulting in changes in GGH localization and protein function.**

**Fig. 3:**
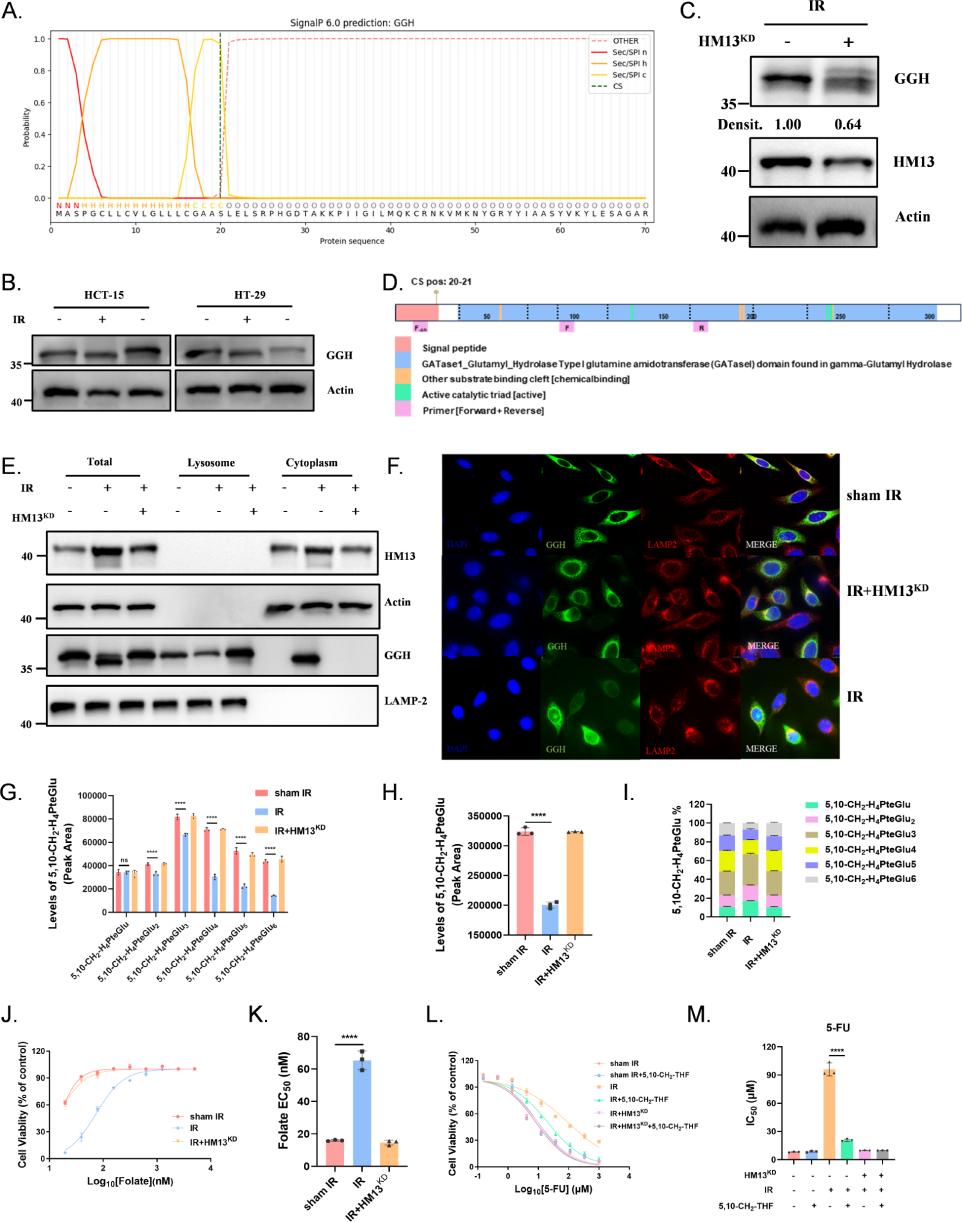
HM13 cleaves the signal peptide, resulting in changes in GGH localization and protein function. (**A**) The binding sites of the GGH gene sequence with signal peptidase were predicted using SignalP 6.0 prediction analysis. (**B**) GGH protein expression in HCT-15 cells. (**C**) GGH protein expression in HM13-knockdown HCT-15 cells. KD: knockdown. (**D**) Schematic diagram of GGH protein domain. (**E**) The total proteins, the proteins localized in lysosomes, the proteins localized in cytoplasm and nucleus were collected by Lysosome isolation experiment. Expression of GGH protein in the lysosomes and cytoplasm of IR group, IR + HM13^KD^ group and sham IR group. (**F**) Localization of GGH protein in IR group, IR + HM13^KD^ group and sham IR group. Red: lysosome; Green: GGH; Blue: DNA. (**G-I**) Levels of 5,10-CH_2_-THF polyglutamated metabolites (5,10-CH_2_-H_4_PteGlu_n_) in various HCT-15 cells. Various types of 5,10-CH_2_-H_4_PteGlu_n_ (**G**); Total 5,10-CH_2_-H_4_PteGlu (**H**); Proportion analysis of various types of 5,10-CH_2_-H_4_PteGlu (**I**). (**J-K**) Multiple gradient folic acid supplementation (maximum concentration 5 mM, 3×gradient dilution) after folic acid deprivation in the various tested groups. Folate growth requirement of IR group, IR + HM13^KD^ group and sham IR group, the dose–response curve: **J**; the bar graph of IC_50_ values: **K**. (**L-M**) Cell viability with 5-FU or 5-FU + 5,10-CH_2_-THF exposure in various HCT-15 cells, the dose–response curve: **L**; the bar graph of IC_50_ values: **M**. All figures were representatives of three independent experiments. Error bars represent the SD. ****P < 0.0001; ns: not significant, two-tailed Student’s t-test

As a crucial signal peptide peptidase, HM13 cleaves the signal sequences (SSs) of secretory precursors, playing an essential role in their maturation (Mentrup et al. 2020). Given the significant 5-FU resistance observed following IR, we used SignaIP 6.0 web software to identify key enzymes in the 5-FU-related metabolic pathway and the 5,10-CH_2_-THF cycle (Fig S3A) (Longley et al. 2003; Teufel et al. 2022; Raz et al. 2016). We found that only GGH carries a signal peptide and associates with sec/spi (Fig. 3A, S3B).

GGH, a key enzyme in the folate metabolism pathway, hydrolyzes polyglutamate tails, reducing intracellular folate pool by converting folate and its derivatives into monomeric forms for cellular export (Wang et al. 2022). Further, compared with sham IR group, the GGH protein band position in IR group moved down (Fig. 3B). It was unexpected to discover that knockdown HM13 after IR caused a significant upward shift in the GGH protein band (Fig. 3C). Previous studies have shown that deletion of the N-terminal signal peptide of GGH leads to a downward shift in the GGH protein band (Yao et al. 1996a, b).We selected appropriate fragments of the signal peptide sequence as the special Forward Primer (F_−ΔN_), and picked two Primer sequences in CDS regions (F, R), which were combined as F_−ΔN_ +R and F + R. We confirmed that GGH signaling peptide fragments are still present when transcribed (Fig. 3D, Fig S4A-C). In general, the loss of signal peptides occurs during the post-translational modification phase.

To confirm changes in GGH protein localization and function, we conducted lysosome isolation experiments. After IR, GGH protein mainly gathered in vitro of lysozyme, while in HM13-knockdown after IR (IR + HM13^KD^) and sham IR groups, GGH protein was primarily localized within the lysosome. (Fig. 3E). Immunofluorescence confirmed the altered localization of GGH protein following IR (Fig. 3F).

We measured the concentrations of polyglutamated folate forms (PteGlu_n_) and 5,10-CH_2_-THF in cells irradiated or IR + HM13^KD^, and discovered that the concentrations of PteGlun and 5,10-CH_2_-THF decreased more rapidly as the number of polyglutamate tails increased after IR, whereas in cells irradiated with IR + HM13KD, the concentrations remained unchanged (Fig. 3G-I, Fig S5A-C). Using methotrexate (MTX) as an exogenous folate analog, we assessed the function of GGH in hydrolyzing polyglutamate tails post-IR. The intracellular levels of polyglutamated MTX, especially those with 5–6 glutamate tails, significantly decreased in IR group (Fig. 3D-F). Using the HM13 inhibitor (Z-LL)_2_ Ketone, we assessed the ability of GGH to hydrolyze polyglutamate tails and found that the hydrolytic ability was decreased. This finding appears to be inconsistent with the HM13 knockdown results, and the underlying mechanism remains unclear. We speculate that this discrepancy may be attributed to either (Z-LL)_2_ Ketone’s unique pharmacological actions or compensatory regulatory pathways that were activated in response to HM13 modulation. (Fig S5G-I).

Cell folate dependency is enhanced due to the reduction of the cellular folate pool, indicating increased GGH protein activity (Fig. 3J-K, Fig S6A-B). Supplementing with 5,10-CH_2_-THF to increase cellular folate pool partially rescued the 5-FU resistance induced by IR. In contrast, adding 5,10-CH_2_-THF to sham IR group or IR + HM13^KD^ group did not decrease the IC_50_ of 5-FU, this indicates that the folate pool may be saturated and unable to further increase the levels of 5,10-CH_2_-THF, thereby not significantly enhancing the formation of the ternary complex or cytotoxicity (Fig. 3L-M).

### Loss of the signal peptide in GGH leads to reduced cellular folate pool and 5-FU-resistance

**Fig. 4:**
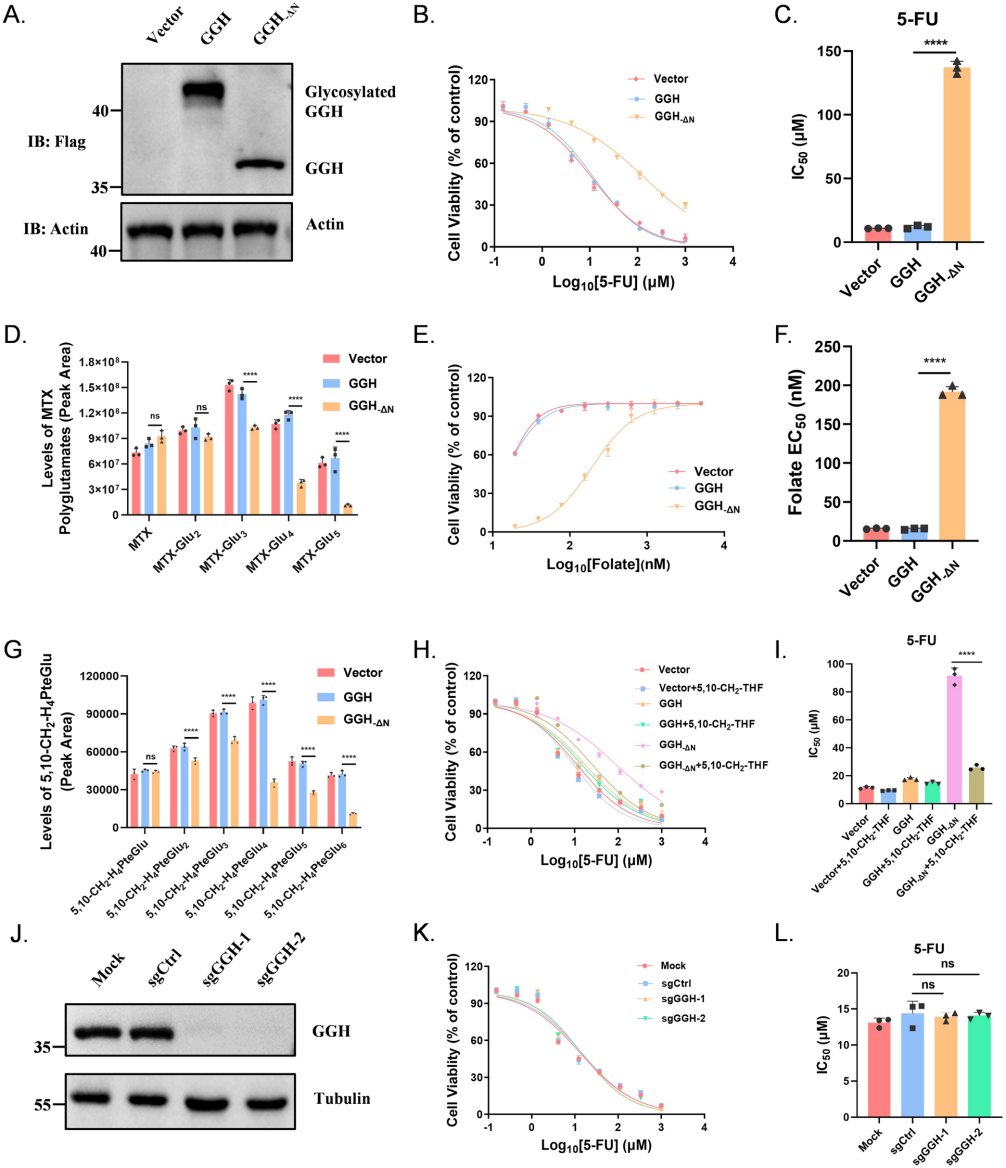
Loss of the signal peptide in GGH leads to reduced cellular folate pool and 5-FU-resistance. (**A**) GGH and GGH_−△N_ overexpression in HCT-15 cells. (**B-C**) Cell viability with 5-FU exposure in various GGH stable expressions of HCT-15 cells, the dose–response curve: B; the bar graph of IC_50_ values: **C**. (**D**) Levels of MTX-polyglutamated metabolites in various GGH overexpression HCT-15 cells. (**E-F**) Folate growth requirements of various GGH stable expression HCT-15 cells, the dose–response curve: **E**; the bar graph of IC_50_ values: **F**. (**G**) Levels of 5,10-CH_2_-THF polyglutamated metabolites (5,10-CH_2_-H_4_PteGlu_n_) in various GGH overexpression HCT-15 cells. (**H-I**) Cell viability with 5-FU or 5-FU + 5,10-CH_2_-THF exposure in various GGH HCT-15 cells, the dose–response curve: **H**; the bar graph of IC_50_ values: **I**. (**J**) GGH protein expression in HCT-15 cells. (**K-L**) Cell viability with 5-FU exposure in HCT-15 cells, the dose–response curve: **K**; the bar graph of IC_50_ values: **L**. All figures were representatives of three independent experiments. Error bars represent the SD. ****P < 0.0001; ns: not significant., two-tailed Student’s t-test

Previous studies have shown that GGH is N-glycosylated when overexpressed directly, whereas removal of the signal peptide results in the loss of glycosylation side chains, which decreases intracellular folate pool, leading to folate-resistance (Wang et al. 2022). We designated the GGH protein lacking the N-terminal signal peptide as “GGH_−ΔN_.” GGH_−ΔN_ elicits the 5-FU resistance of HCT-15 (Fig. 4A-C). Moreover, using exogenous MTX as a folate analog to assess the hydrolytic capability of GGH_−ΔN_, we observed enhanced hydrolysis of polyglutamate tails (Fig. 4D). Therefore, we hypothesized that the resistance to 5-FU because of the enhanced hydrolysis ability of GGH, resulting in a decrease of the concentration of 5,10-CH_2_-THF.

Furthermore, cells expressing GGH without the signal peptide exhibited increased dependence on folate, indicating reduced intracellular folate pool (Fig. 4E-F). And we measured the concentrations of 5,10-CH_2_-THF within the cells overexpressing GGH_−ΔN_ and discovered that the results were consistent with the high expression of HM13 (Fig. 4G). Drug sensitivity assays with 5,10-CH_2_-THF supplementation showed that increasing cellular folate levels could partially rescue 5-FU resistance when GGH lacked the signal peptide, consistent with the results shown in Fig. 3L (Fig. 4H-I).

Moreover, the cellular folate pool of HCT-15 cells with GGH knockdown was not significantly increased upon 5-FU treatment. Likewise, the hydrolytic capacity of polyglutamated folate analogs remained unchanged. This indicates the existence of other polyglutamate hydrolases in the cell (Fig. 4J-L, S6C-E).

### Met enhances the cytotoxic effect of 5-FU by mediating cellular folate pool

**Fig. 5:**
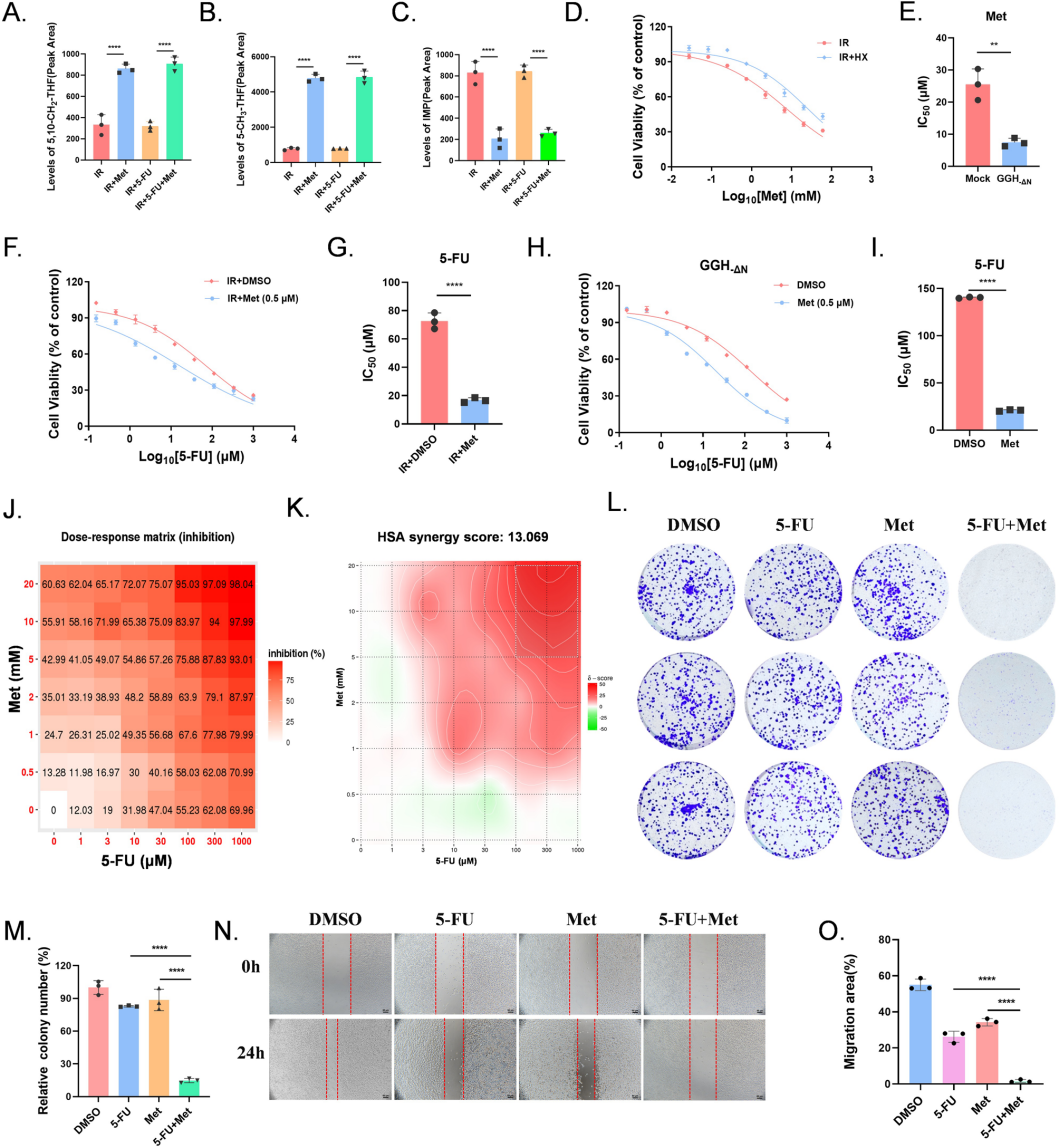
Met enhances the cytotoxic effect of 5-FU by increasing cellular folate pool. (**A-C**) The levels of 5,10-CH_2_-THF, 5-CH_3_-THF and IMP in HCT-15 cells were measured following treatment with 5-FU, Met, or 5-FU + Met. (**D-E**) Cell viability with Met or Met + Hypoxanthine (100µM) exposure in IR group, the dose–response curve: **D**; the bar graph of IC_50_ values: **E**. (**F-I**) Cell viability with 5-FU exposure in medium containing Met (0.5 mM) in IR group or GGH_−△N_ stable expressions of HCT-15 cells., the dose–response curve: **F, H**; the bar graph of IC_50_ values: **G, I**. (**J-K**) The combined treatment of 5-FU (0, 1, 3, 10, 30, 100, 300 and 1000 µM) and Met (0, 0.5, 1, 2, 5, 10 and 20 mM) were assessed in the irradiation group (2 Gy × 8) HCT-15 cells. The HSA synergy scores was visualized using the SynergyFinder. (**L-M**) HCT-15 cells after irradiated 2 Gy × 8 using/nonusing 5-FU, Met, 5-FU + Met, were subjected to colony formation assay (**L**), quantification data (**M**), *n* = 3. (**N-O**) HCT-15 cells after irradiated 2 Gy × 8 using/nonusing 5-FU, Met, 5-FU + Met, were subjected to wound healing assay (**O**) and results were quantified (**N**), *n* = 3. All figures were representatives of three independent experiments. Error bars represent the SD, ns: not significant; **P < 0.01, ****P < 0.0001; two-tailed Student’s t-test

Studies manifested that Met impairs cellular one-carbon metabolism in a manner similar to the antifolate drugs, leading to the accumulation of folate pathway metabolites, and reducing one-carbon unit transfer in the folate cycle, thereby decreasing purine biosynthesis (Corominas-Faja et al. 2012; Janzer et al. 2014). Our LC-MS analysis revealed using Met significantly increased the biosynthesis of 5,10-CH_2_-THF and 5-CH_3_-THF (Fig. 5A-B). Furthermore, Met significantly reduced intracellular inosine monophosphate (IMP) concentration (Fig. 5C). Indeed, the cytotoxic effects of Met could be effectively rescued by HX supplementation, indicating a decrease in purine biosynthesis which is consistent with previous studies (Fig. 5D-E) (Corominas-Faja et al. 2012; Jara and Lopez-Munoz 2015). The current study revealed that Met disturbs the homeostasis of the folic acid cycle in C. elegans, leading to the increasing levels of 5-CH_3_-THF and 5,10-CH_2_-THF, while decreasing THF levels (Cabreiro et al. 2013). Moreover, it can increase SAM and SAH levels while decreasing methionine levels by blocking methionine synthase (MS). We validated this finding in HCT-15 cells, elucidating the mechanism of action of 5-FU and Met in the treatment of colon cancer (Fig S7A-E).

Meanwhile, we assessed the IC_50_ values of 5-FU after the use of Met (0.5mM) after IR, which were significantly reduced by Met (Fig. 5F-G). Similar results were found in cells overexpressing GGH_−ΔN_ (Fig. 5H-I). We conducted drug sensitivity assays to further investigate the synergistic effect of Met and 5-FU. And the highest single agent (HSA) score was 13.069, indicating an obvious synergistic effect. Collectively, which indicate that Met amplifies the cytotoxicity of 5-FU in cells after IR (Fig. 5J-K). Furthermore, 5-FU + Met substantially inhibited colony formation in HCT-15 cells after IR (Fig. 5L-M). Similarly, 5-FU + Met substantially inhibited wound healing in HCT-15 cells after IR (Fig. 5N-O). In conclusion, these results establish the central role of 5-FU + Met in CRC progression.

### 5-FU-resistance can be overcome by the combination of Met in vivo

**Fig. 6:**
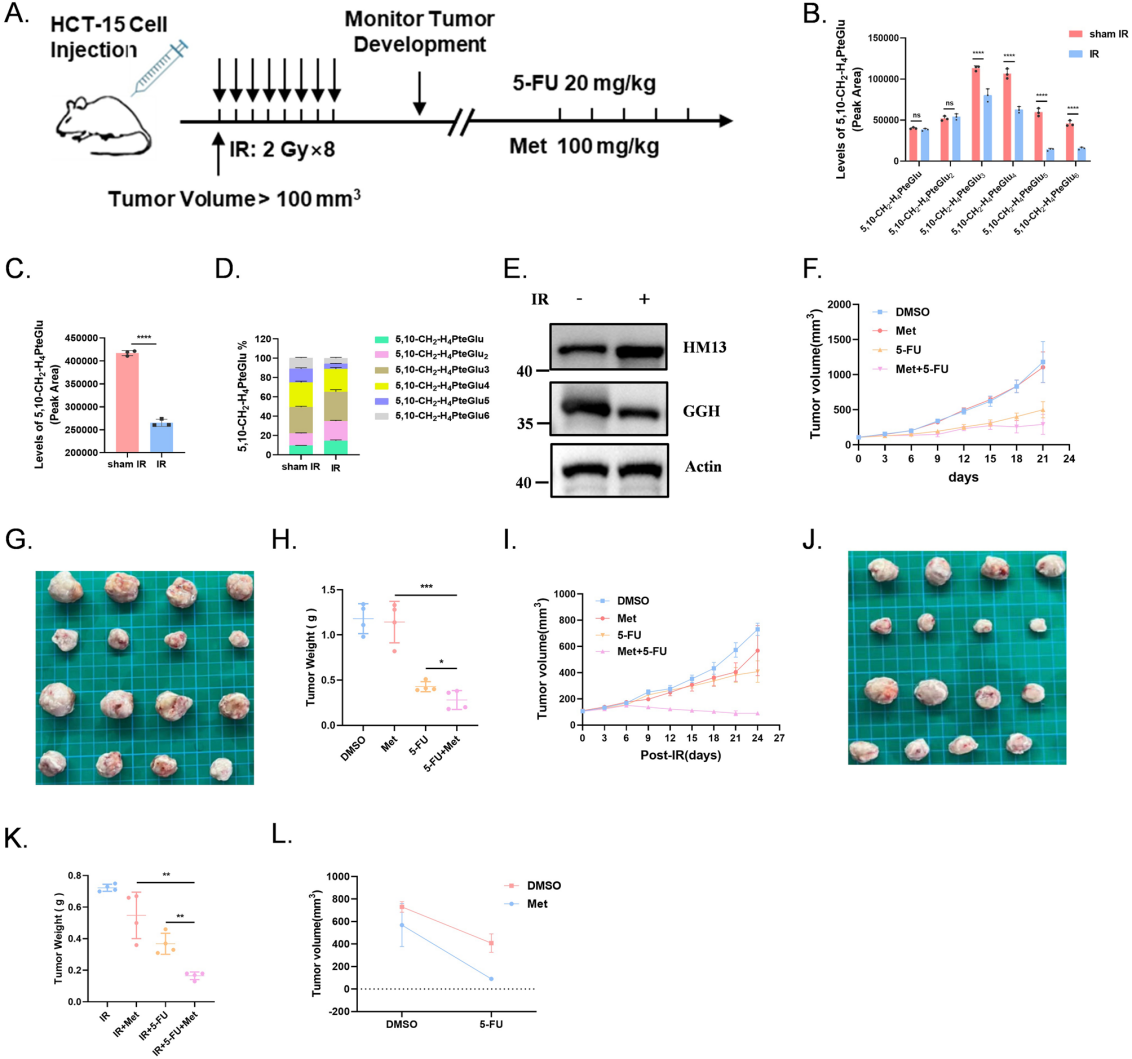
5-FU-resistance can be overcome by the combination of Met in vivo. (**A**) Scheme for 5-FU, Met, and combination therapy after irradiation treatment in the HCT-15-injection nude mouse. (**B-D**) Levels 5,10-CH_2_-H_4_PteGlu_n_ in IR group and sham IR group HCT-15-injection nude mouse. Various types of 5,10-CH_2_-H_4_PteGlu_n_ (**B**); Total 5,10-CH_2_-H_4_PteGlu (**C**); Proportion analysis of various types of 5,10-CH_2_-H_4_PteGlu (**D**). (**E**) Endogenous GGH and HM13 protein expression in IR group, IR + HM13^KD^ group and sham IR group HCT-15-injection nude mouse. (*n* = 4). (**F-H**) Growth curve (**F**) and photographs (**G**) of tumors collected from mice treated with 5-FU, Met or 5-FU + Met; Tumor weight at the endpoint of the in vivo experiment (**H**). (**I-K**) Growth curve (**I**) and photographs (**J**) of tumors collected from mice-irradiated (2 Gy × 8) treated with 5-FU, Met and the combination; Tumor weight at the endpoint of the in vivo experiment (**K**). (*n* = 4). (**L**) Effect of 5-FU and Met combined therapy (post-IR Day 24). Error bars represent the SD. *P < 0.05, **P < 0.01, ***P < 0.001, ****P < 0.0001, two-tailed Student’s t-test

The subcutaneous HCT-15 tumor model was established in nude mouse, and which were divided into sham IR group and IR (Fig. 6A). The IR group was further divided into several subgroups: sham Drug, 5-FU, Met, and 5-FU combined with Met (Fig. 6A).

First, we assessed the content of 5,10-CH_2_-THF in tumor tissues from mice irradiated with 2 Gy × 8, and the content of 5, 10-CH_2_-THF was significantly reduced in tumor tissues, consistent with our in vitro results (Fig. 6B-D). Western blot analysis confirmed increased HM13 expression, and led to a downward shift in the GGH protein band in IR group (Fig. 6E). We found that there was no obvious synergistic effect between 5-FU and Met in sham IR group. (Fig. 6F-H, Fig S8A-B). In contrast, our results showed that 5-FU + Met significantly blocked CRC growth in vivo after the indicated IR which corroborated the in vitro findings (Fig. 6I-L, Fig S8C). The possible reason is that the GGH protein in sham IR group functioned normally and was located in the lysosome. There is sufficient 5,10-CH_2_-THF in cells to combine with FdUMP, to form ternary complexes, so the supply of folate pool provided by metformin does not effectively increase the cytotoxicity of 5-FU.

## Discussion

Despite recent advancements in the treatment of CRC, there has been no substantial improvement in long-term survival rates or prognosis. Hence, combining targeted therapies with surgical and radiotherapy is vital for improving the prognosis of CRC patients.

5-FU sensitizes CRC cells to irradiation (Shewach and Lawrence 2007). However, increasing evidence indicates 5-FU may be noneffective after irradiation. Therefore, it is critical to investigate the potential resistance mechanism of 5-FU after irradiation in order to guide the clinical curative effect, ultimately improving the prognosis of patients with CRC.

We performed RNA-seq on CRC cell lines irradiated and on 5-FU resistance cell lines and identified HM13 overactivation as a key influencing factor. Recent studies have reported that elevated HM13 expression correlates with poor prognosis in lung cancer, hepatocellular carcinoma, breast cancer, and glioblastoma patients (Wei et al. 2017; Zhang et al. 2022; Zhou et al. 2021; Yang et al. 2022). Further investigation of HM13 protein function and related signaling pathways should help to determine whether HM13 can be used as a potential biomarker to predict 5-FU chemotherapy resistance in colorectal cancer.

Studies on 5-FU resistance continues to be a central focus. Recently, several studies have reported that Nano-formulated drug delivery of 5-FU can enhance the therapeutic effect of 5-FU. Folic-acid conjugated liposomes of 5-FU might increase the therapeutic efficacy of drug whereas the toxic side effects have been decreased; the nanoscale fluoropyrimidine polymer (CF10) might be effective at inhibiting CRC metastatic progression (Sah et al. 2023; Sethy and Kundu 2021). In addition, strategies targeting cell surface or intracellular receptors are emerging as valuable approaches to reverse existing resistance mechanisms (Sah et al. 2024). Given that 5-FU is an antimetabolite drug and HM13 is involved in the maturation of metabolically relevant exoenzymes, we focused on the lesser-studied 5,10-CH_2_-THF in the ternary complex, a crucial component of the intracellular folate cycle. We evaluated key enzymes in the folate cycle and found that only the GGH gene contains an N-terminal signal peptide. Further analysis revealed that abnormal deletion of the GGH signal peptide forms the GGH_−ΔN_ protein, causing GGH to localize in the cytoplasm, consistent with our previous research. Normally, GGH resides in lysosomes, where its activity is limited by lysosomal membrane transport rates, but GGH-_ΔN_ exerts its hydrolytic function directly in the cytoplasm, significantly enhancing GGH activity. This results in the hydrolysis and cellular expulsion of polyglutamylated 5,10-CH_2_-THF, lowering intracellular folate levels, reducing the overall folate cycle, and decreasing 5,10-CH_2_-THF production. Which hinders complex formation, leading to 5-FU resistance.

The methyl trap hypothesis (5,10-CH_2_-THF’s irreversible transformation into 5-CH_3_-THF) suggests that Met could promote the accumulation of folate in the direction of 5-CH_3_-THF (Mato et al. 2008). We also found that there was an increase in the content of 5, 10-CH_2_-THF. Therefore, Met can promote the formation of 5-FU ternary complex and enhance its cytotoxic effect. Met affects glucose metabolism and purine metabolism, and 5-FU affects DNA and RNA biosynthesis. The combination of Met and 5-FU can lead to the destruction of various metabolic homeostasis in cells and accelerate the apoptosis of tumor cells. However, the specific pathways by which metformin affects intermediates of folate metabolism remain unclear.

Furthermore, our study primarily used HCT-15 cells as an experimental model, which may limit the broader applicability of our findings. To establish more comprehensive clinical relevance, subsequent studies should include validation of other CRC molecular subtypes and patient-derived samples. In addition, although our data suggest that metformin and 5-FU synergistically work well in irradiated HCT-15 cells and nude mouse models, the potential toxicity of this combination regimen remains to be further evaluated. Additionally, the direct cause of HM13 elevation following conventional fractionated irradiation warrants further investigation to more effectively address potential 5-FU resistance post-IR, our previous study also identified gene mRNA expression differences between single high-dose and fractionated radiotherapy in HCC (Zhao et al. 2023). Therefore, we hypothesized that the mRNA expression of HM13 may be related to the irradiation frequency, indicating the need for further research on the different effects of activation of signaling pathways, cell metabolism and the immune microenvironment of varying dose fractionations under equivalent BED conditions.

We revealed that IR drives an axis of 5-FU resistance (HM13-GGH-5,10-CH_2_-THF axis), and that Met increases the intracellular 5,10-CH_2_-THF concentration, which enhances the cytotoxic effects of 5-FU, reveal a new mechanism by which metformin promotes the efficacy of 5-FU, helping to expand the indication of metformin. Therefore, we propose a strategy to counteract 5-FU resistance following irradiation and demonstrate the potential mechanism, providing guidance for post-radiotherapy chemotherapy regimens.

## Conclusion

Our study suggests that the HM13-GGH-5,10-CH_2_-THF axis leads to 5-FU resistance after IR (2 Gy × 8), and the combination therapy of Met and 5-FU may be a potential effective method to address drug resistance after radiotherapy. In summary, our study will help to reveal the mechanisms of IR-induced 5-FU resistance, which is important for finding new therapeutic targets and improving the efficacy of chemotherapy regimens after radiotherapy.

## Electronic supplementary material

Below is the link to the electronic supplementary material.

Supplementary Material 1

## Data Availability

The RNA-seq data generated in this study is available in SRA under accession number PRJNA1197681. Other data that support the findings of this study are available from the corresponding author upon request (zeng.zhaochong@zs-hospital.sh.cn). Method section described all the data used for analysis in this study.

## Abbreviations

CRC: Colorectal cancer
DDR: DNA damage repair
dNTP: Deoxynucleoside triphosphate
dTMP: Deoxythymidine monophosphate
dTTP: Deoxythymidine triphosphate
dUMP: 2′-deoxyuridine-5′-monophosphate
dUTP: Deoxyuridine triphosphate
FdUMP: 5-fluoro-2’-deoxyuridylate monophosphate
GGH: γ-Glutamyl Hydrolase
GTEx: Genotype-Tissue Expression
HM13: Histocompatibility Minor 13
HSA: Highest single agent
IC_50_: The half-maximal inhibitory concentration
IMP: Inosine monophosphate
LV: Leucovorin
Met: Metformin
MTX: Methotrexate
OS: Overall survival
RNA-seq: RNA-sequencing
RT: Radiation therapy
SSs: Signal sequences
TCGA: The Cancer Genome Atlas
TS: Thymidylate synthase
5-CH_3_-THF: 5-methyl-tetrahydrofolate
5-FdUTP: 5-fluoro-2’-deoxyuridine triphosphate
5-FU: 5-fluorouracil
5,10-CH_2_-THF: 5, 10-methylenetetrahydrofolate
