# Supplementary material for: Metformin reverses 5-FU resistance induced by radiotherapy through mediating folate metabolism in colorectal cancer
Source: Mol Med. 2025 May 21;31:199. doi: 10.1186/s10020-025-01206-5 (PMC12093704; doi:10.1186/s10020-025-01206-5)
Supplement: Supplementary file 1 — Supplementary Material 1 [file 10020_2025_1206_MOESM1_ESM.docx]

**Supplemental materials**

**Metformin reverses 5-FU resistance induced by radiotherapy through mediating folate metabolism in colorectal cancer**

Shuxuan Wang^1,2*^, Yanyan Lin^1,2*^, Qianqian Zhao^1,2^, Huanliang Chen^1,2^, Shisuo Du^1,2^, Zhaochong Zeng^1,2#^

^1^Department of Radiation Oncology, Zhongshan Hospital, Fudan University, Shanghai, 200032, China

^2^Cancer Center, Zhongshan Hospital, Fudan University, Shanghai, 200032, China

^*^Shuxuan Wang, Yanyan Lin contributed equally to this study.

^#^Correspondence: Zhaochong Zeng, Dept. of Radiation Oncology, 180 Fenglin Road, Zhongshan Hospital, Fudan University, Shanghai, 200032, China, e-mail: zeng.zhaochong@zs-hospital.sh.cn.

**Supplemental Methods**

mRNA sequencing experimental method

RNA Isolation and Library Preparation:

Total RNA was extracted using the TRIzol reagent (Invitrogen, CA, USA) according to the manufacturer’s protocol. RNA purity and quantification were evaluated using the NanoDrop 2000 spectrophotometer (Thermo Scientific, USA). RNA integrity was assessed using the Agilent 2100 Bioanalyzer (Agilent Technologies, Santa Clara, CA, USA). Then the libraries were constructed using VAHTS Universal V6 RNA-seq Library Prep Kit according to the manufacturer’s instructions. The transcriptome sequencing and analysis were conducted by OE Biotech Co., Ltd. (Shanghai, China).

RNA Sequencing and Differentially Expressed Genes Analysis:

The libraries were sequenced on an Illumina Novaseq 6000 platform and 150 bp paired-end reads were generated. About 39.09 M~49.73 M raw reads for each sample were generated (Table S1). Raw reads of fastq form at were firstly processed using fastp and the low-quality reads were removed to obtain the clean reads^1^. Then about 37.48 M - 47.25 M clean reads for each sample were retained for subsequent analyses. The clean reads were mapped to the reference genome using HISAT2^2^. FPKM of each gene was calculated and the read counts of each gene were obtained by HTSeq-count^3,4^. PCA analysis were performed using R (v 3.2.0) to evaluate the biological duplication of samples. Differential expression analysis was performed using the DESeq2^5^. Q value < 0.05 and foldchange > 2 or foldchange < 0.5 was set as the threshold for significantly differential expression gene (DEGs). Hierarchical cluster analysis of DEGs was performed using R (v 3.2.0) to demonstrate the expression pattern of genes in different groups and samples. The radar map of top 30 genes was drawn to show the expression of up-regulated or down-regulated DEGs using R packet ggradar.

**Reference:**

1. Chen S, Zhou Y, Chen Y, Gu J. fastp: an ultra-fast all-in-one FASTQ preprocessor. *Bioinformatics*. Sep 1 2018;34(17):i884-i890. doi:10.1093/bioinformatics/bty560

2. Kim D, Langmead B, Salzberg SL. HISAT: a fast spliced aligner with low memory requirements. *Nat Methods*. Apr 2015;12(4):357-60. doi:10.1038/nmeth.3317

3. Anders S, Pyl PT, Huber W. HTSeq--a Python framework to work with high-throughput sequencing data. *Bioinformatics*. Jan 15 2015;31(2):166-9. doi:10.1093/bioinformatics/btu638

4. Roberts A, Trapnell C, Donaghey J, Rinn JL, Pachter L. Improving RNA-Seq expression estimates by correcting for fragment bias. *Genome Biol*. 2011;12(3):R22. doi:10.1186/gb-2011-12-3-r22

5. Love MI, Huber W, Anders S. Moderated estimation of fold change and dispersion for RNA-seq data with DESeq2. *Genome Biol*. 2014;15(12):550. doi:10.1186/s13059-014-0550-8

**Supplemental Figures and Figure legends**


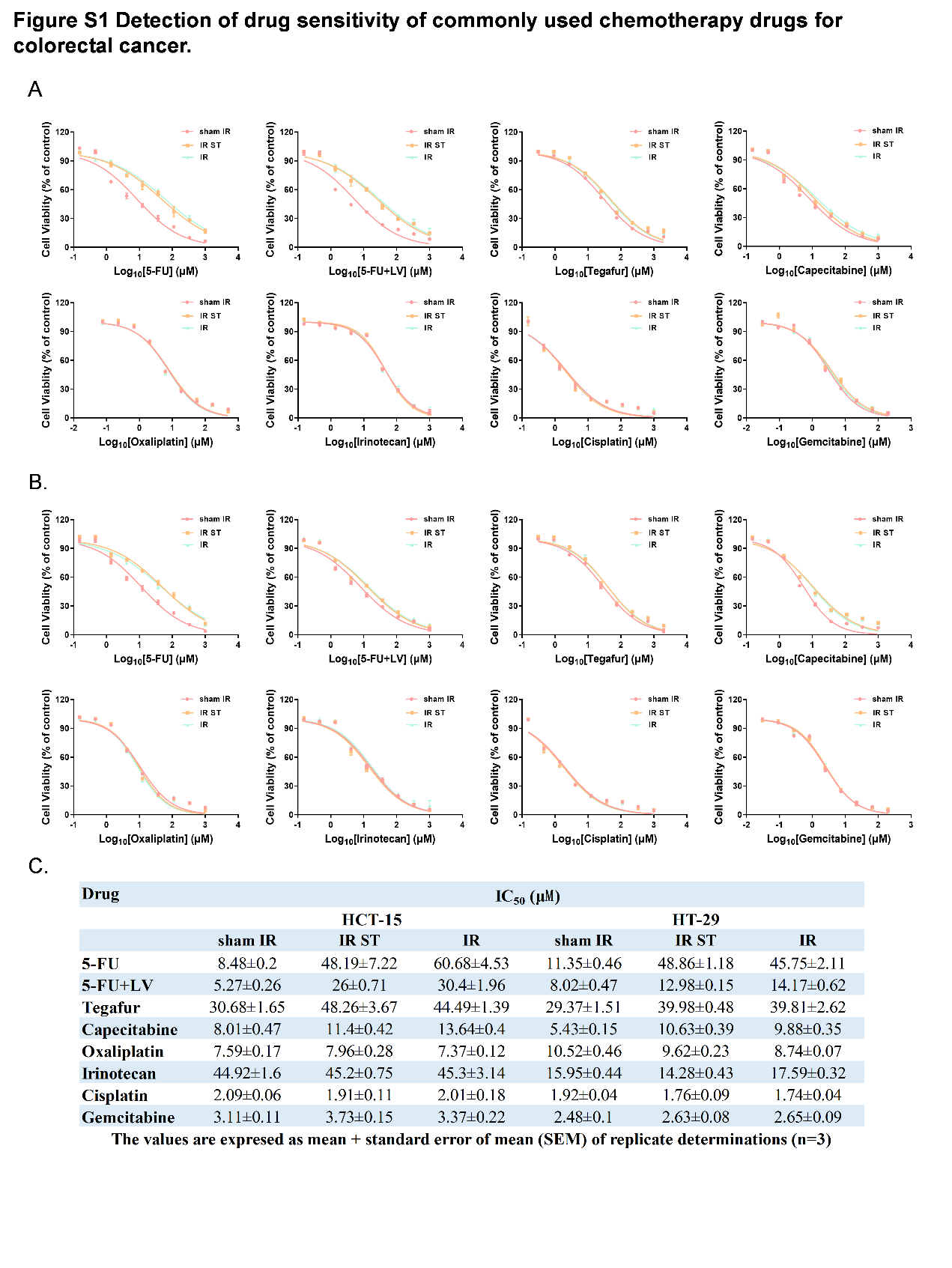


**Fig S1**

Detection of drug sensitivity of commonly used chemotherapy drugs for colorectal cancer. (A-B) Cell viability with 5-FU, 5-FU+LV, Tegafur, Capecitabine, Oxaliplatin, Irinotecan, Cisplatin, and Gemcitabine exposure in various HCT-15 (A) or HT-29 (B) cells. Data is presented as a dose–response curve. (C) Table of IC_50_ of multiple drugs for HCT-15 and HT-29 cell lines after IR.


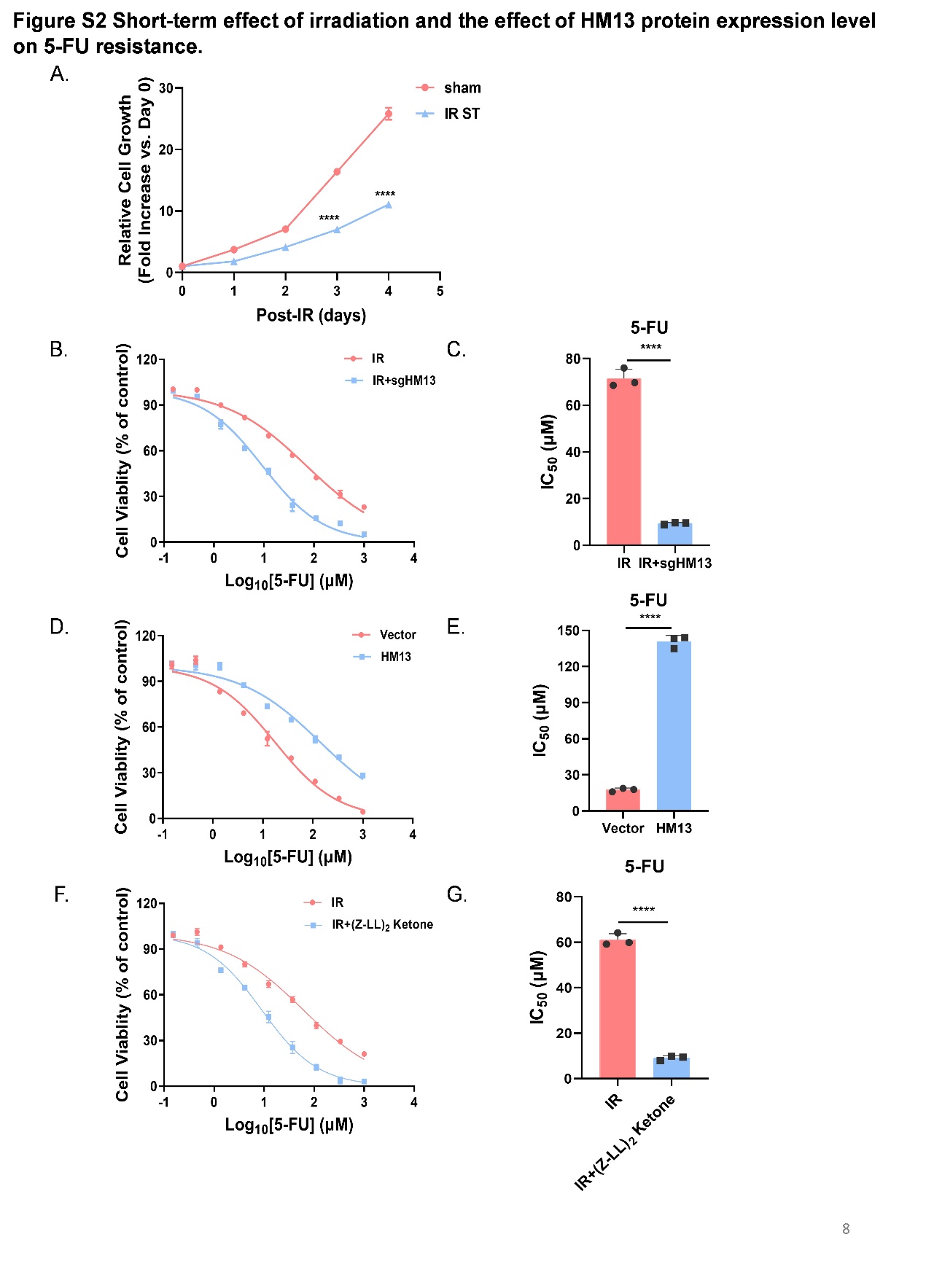


**Fig S2**

Short-term effect of irradiation and the effect of HM13 protein expression level on 5-FU resistance. (A) Growth curves of IR ST groups and sham IR groups. Data are presented as means ±SD. (B-C) Cell viability with 5-FU exposure in irradiated HT-29 cells and sgHM13 irradiated HT-29 cells, the dose–response curve: B; the bar graph of IC_50_ values: C. (D-E) Cell viability in response to pulse exposure of 5-FU in parental and exogenous overexpression of HM13 HT-29 cells. Data are shown as dose–response curve (D) and bar graph of IC_50_ values (E). (F-G) Cell viability with 5-FU exposure in HM13-inhibited HT-29 cells after IR , the dose–response curve: F; the bar graph of IC_50_ values: G. All figures were representatives of three independent experiments. Error bars represent the SD. ****P < 0.0001, two-tailed Student’s t-test.


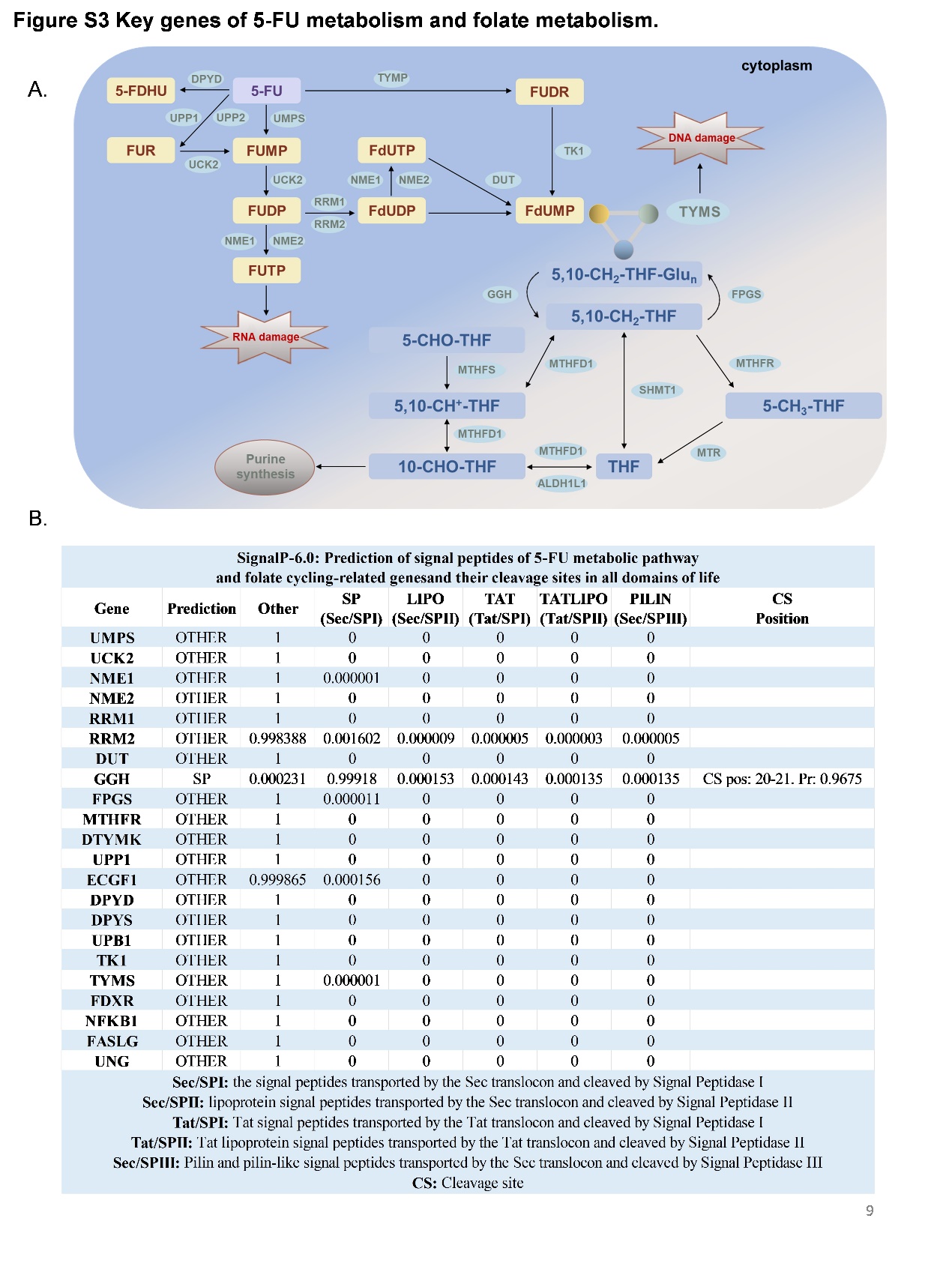


**Fig S3**

Key genes of 5-FU metabolism and folate metabolism. (A) Schematic diagram of folate metabolism and 5-FU metabolism in the cytoplasm. (B) Signal peptide prediction of genes related to 5-FU metabolism and folate cycling.


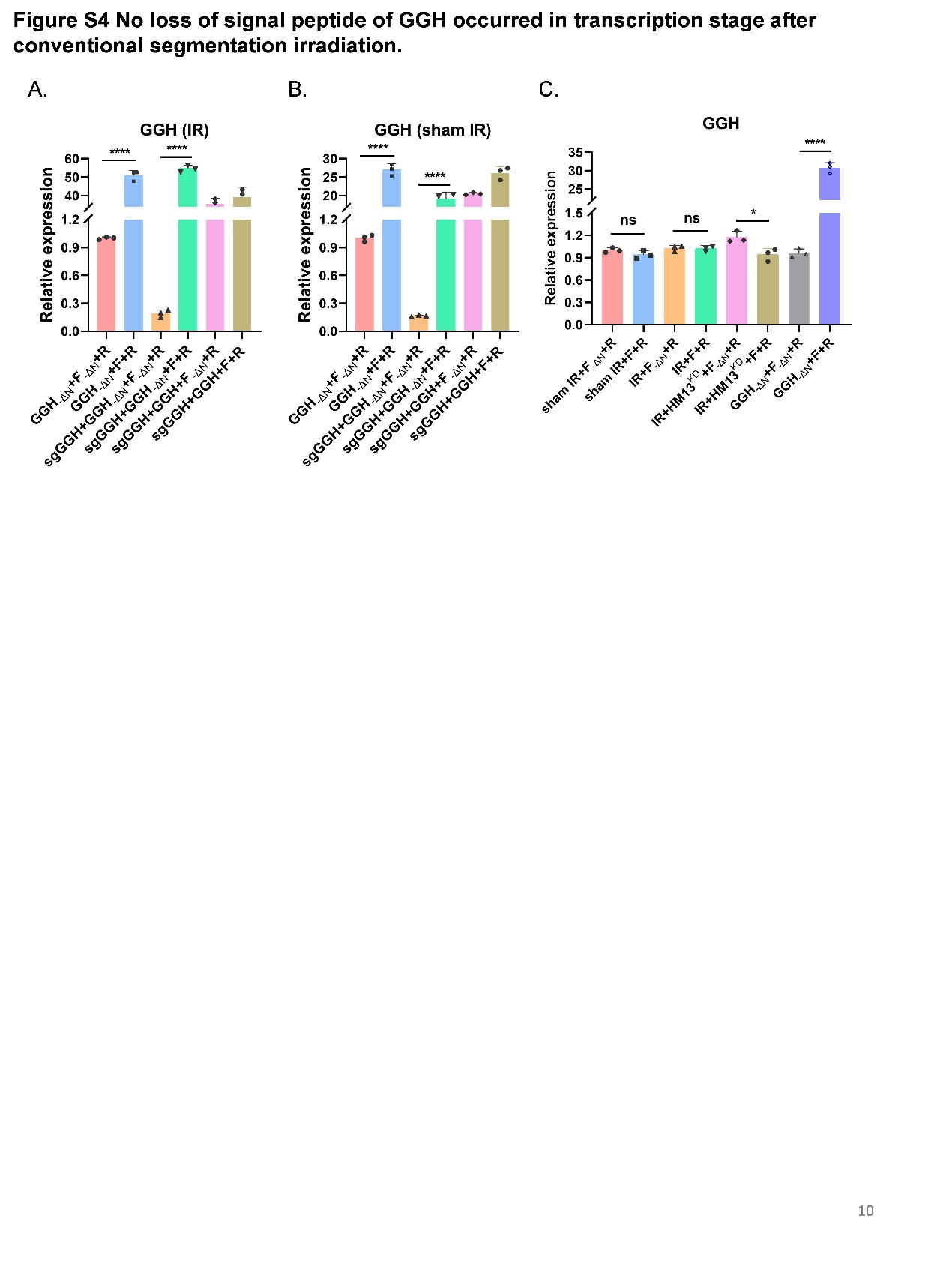


**Fig S4**

No loss of signal peptide of GGH occurred in transcription stage after conventional segmentation irradiation. (A-C) The relative mRNA levels of GGH in HCT-15 cells of various GGH types (GGH, GGH_-ΔN_, sgGGH+ GGH_-ΔN_) after irradiation (2 Gy × 8) were determined by RT-qPCR. F_-ΔN_: Forward Primer, the primer sequence is located in the signal peptide. F: Forward Primer. R: Reverse Primer. All figures were representatives of three independent experiments. Error bars represent the SD. *P < 0.05, ****P < 0.0001, two-tailed Student’s t-test.


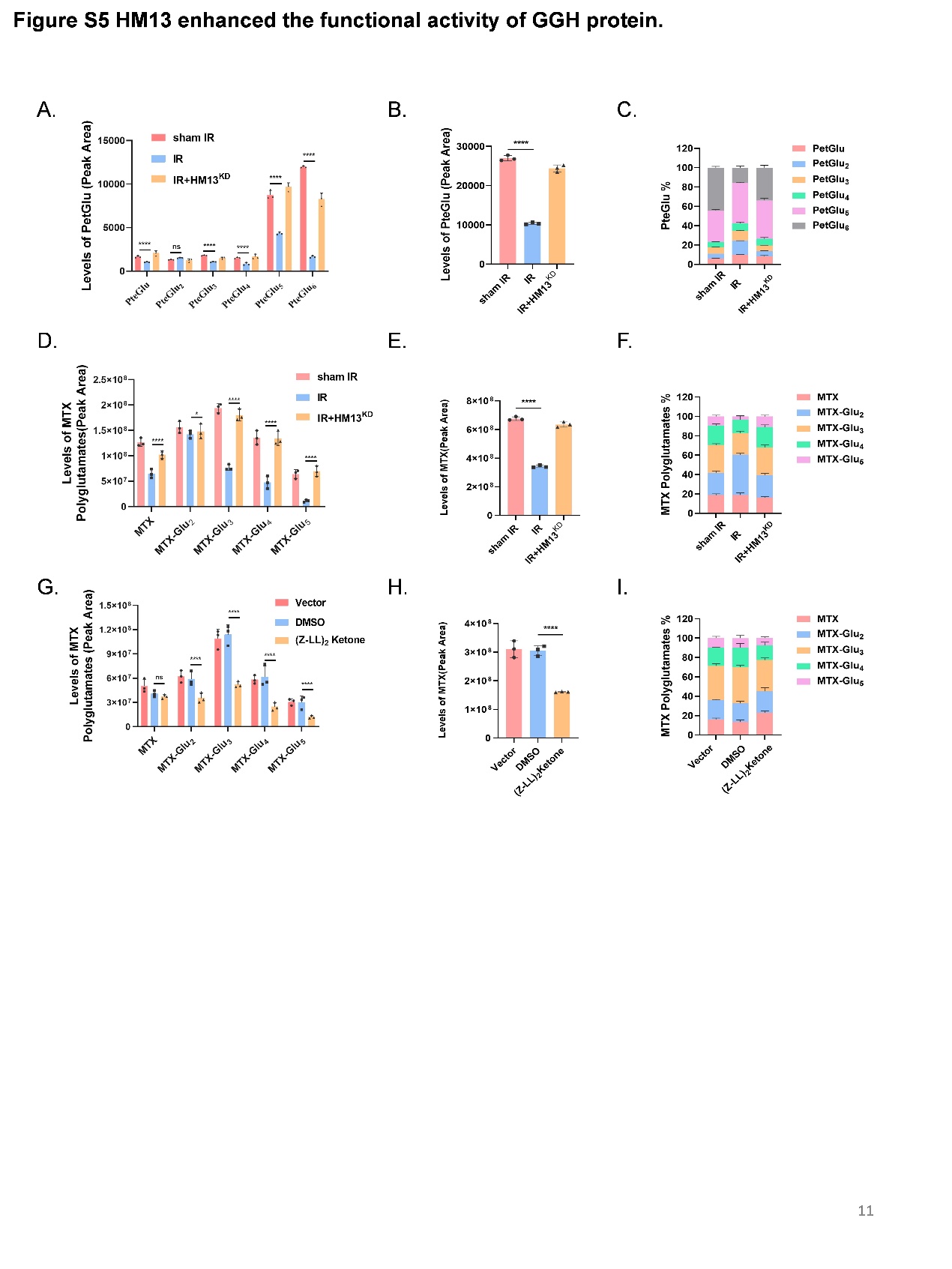


**Figure S5**

(A-C) Levels of THF polyglutamated metabolites (PteGlu) in various HCT-15 cells. Various types of PteGlun (A); Total PteGlu (B); Proportion analysis of various types of PteGlu (C). (D-F) Levels of MTX-polyglutamated metabolites in HCT-15 cells. Various types of MTX polyglutamated (D); Total MTX (E); Proportion analysis of various types of MTX polyglutamated (F). (G-I) Levels of MTX-polyglutamated metabolites in HCT-15 cells after HM13 inhibitor (Z-LL)_2_ ketone treatment. Various types of MTX polyglutamated (G); Total MTX (H); Proportion analysis of various types of MTX polyglutamated (I).


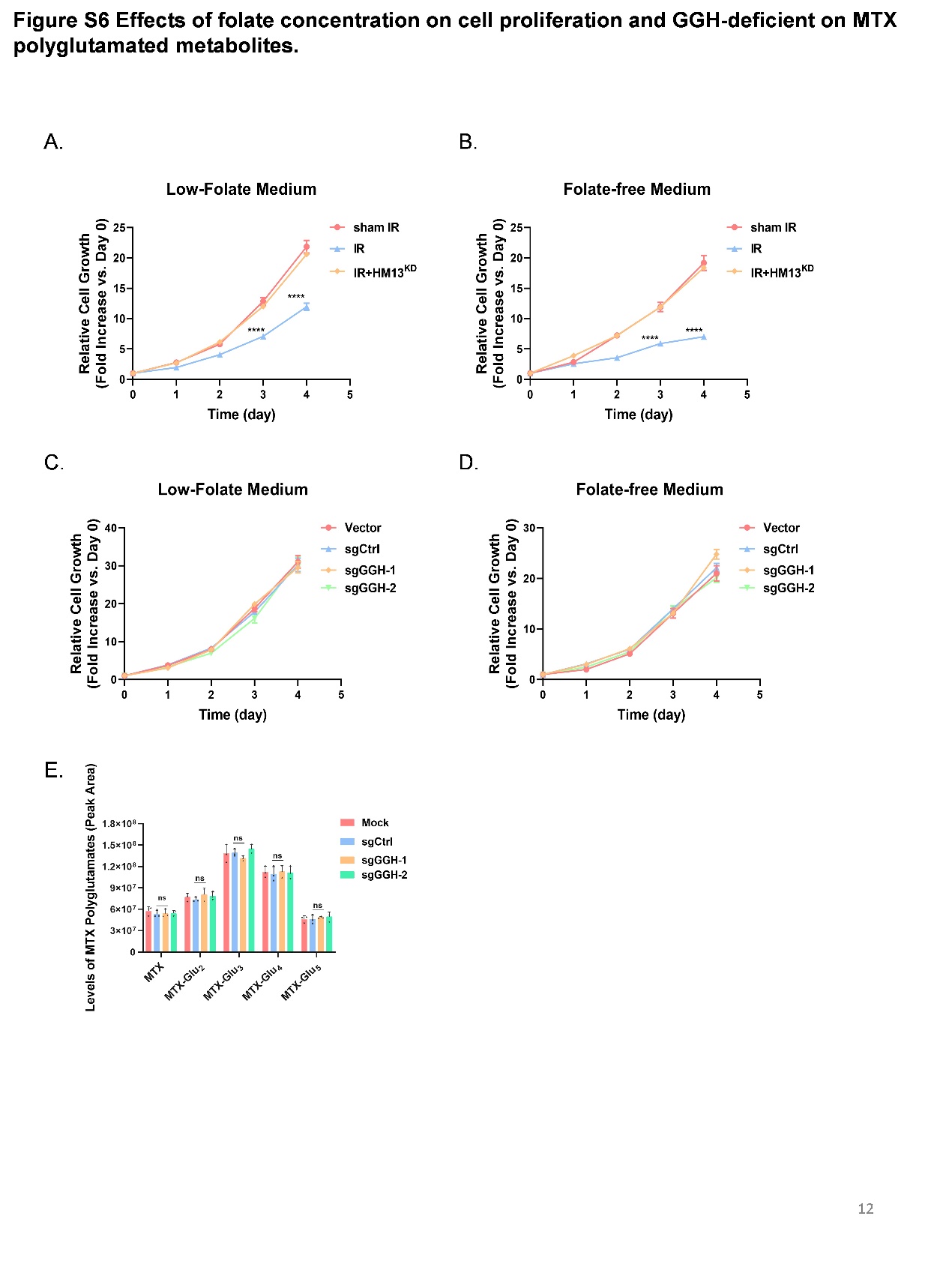


**Fig S6**

Effects of folate concentration on cell proliferation and GGH-deficient on MTX polyglutamated metabolites. (A-B) Growth curves of HCT-15 cells IR or IR+HM13^KD^ under different folate concentration conditions. (C-D) Growth curves of sgCtrl and sgGGH in HCT-15 cells under different folate concentration conditions. (E) Levels of MTX-polyglutamated metabolites in sgCtrl and sgGGH HCT-15 cells. All figures were representatives of three independent experiments. Error bars represent the SD. ****P < 0.0001, two-tailed Student’s t-test.


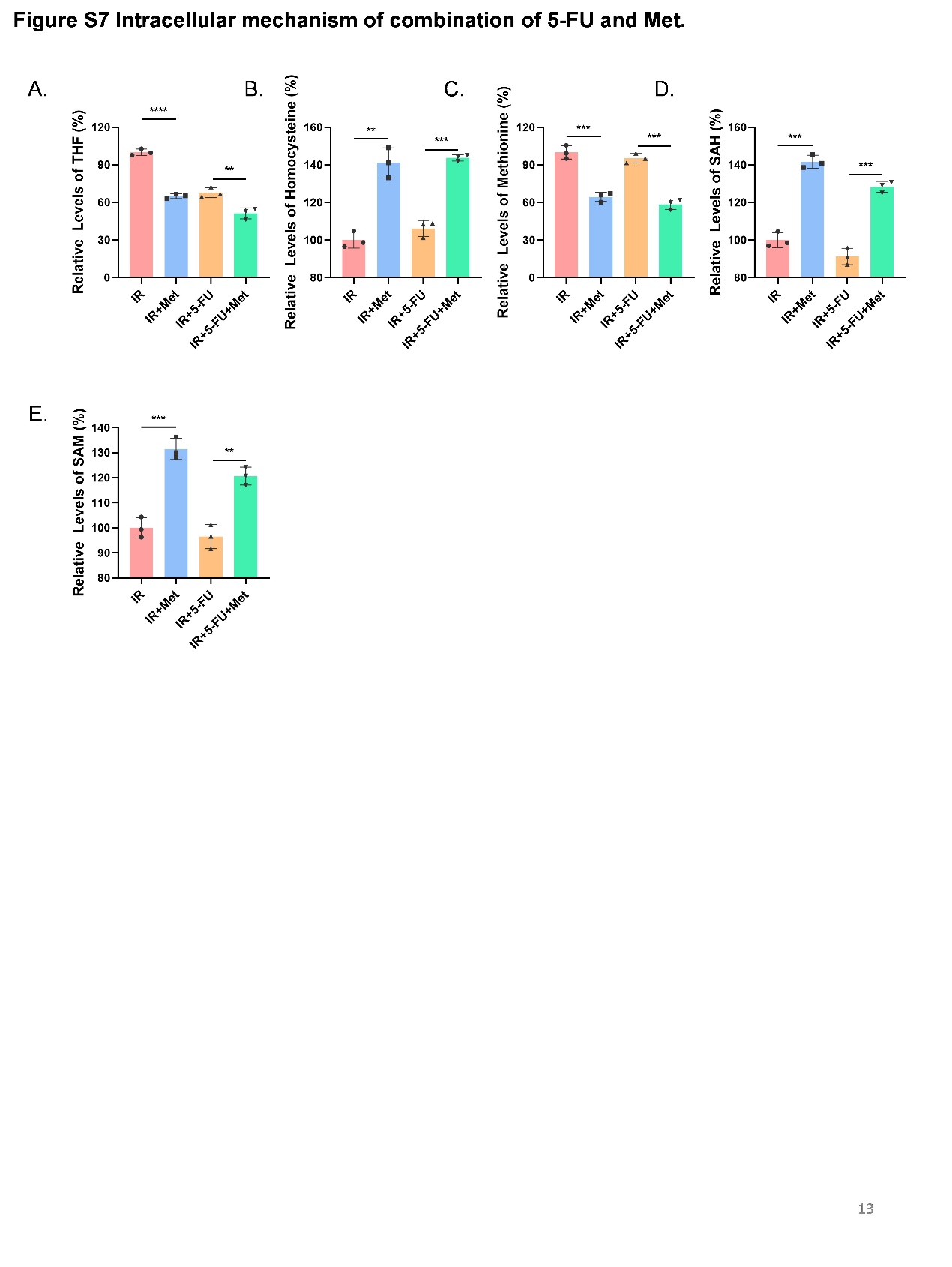


**Fig S7**

Intracellular mechanism of combination of 5-FU and Met. (A-E) The levels of THF (A), Homocysteine (B), Methionine (C), SAH (D) and SAM (E) in HCT-15 cells were measured following treatment with 5-FU, Met, or the combination of 5-FU and Met after IR. All figures were representatives of three independent experiments. Error bars represent the SD, two-tailed Student’s t-test.


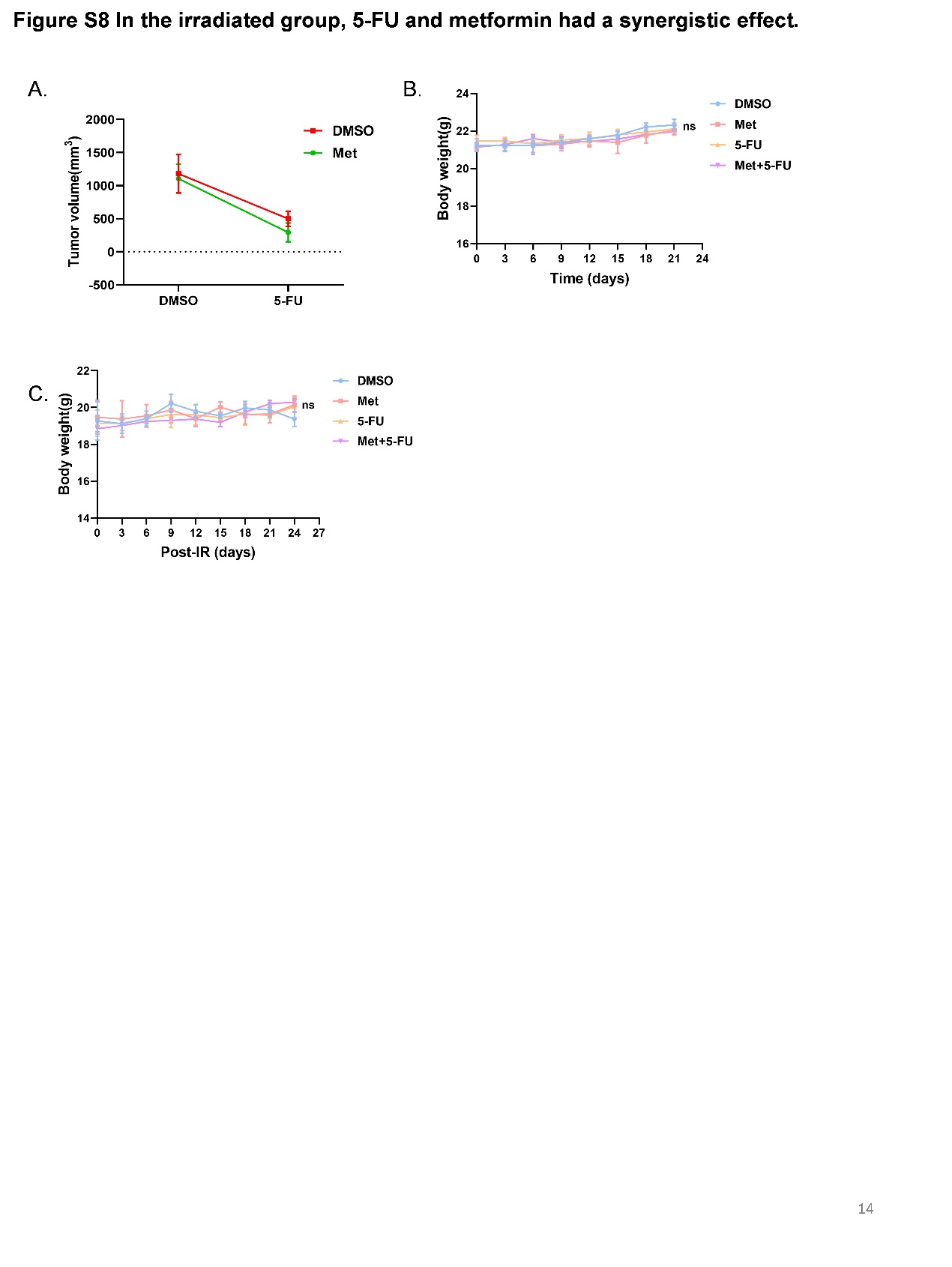


**Fig S8**

In the irradiated group, 5-FU and metformin had a synergistic effect. (A) Effect of 5-FU and Met combined therapy in sham IR group (post-therapy Day 21). (B) The body weight at the indicated time of the in vivo Met, 5-FU, Met+5-FU treatment experiment. (C) The body weight at the indicated time of the in vivo after the indicated irradiation (2 Gy × 8) using Met, 5-FU, Met+5-FU treatment experiment. Error bars represent the SD, two-tailed Student’s t-test.
